# Integrated biclustering of heterogeneous genome-wide datasets for the inference of global regulatory networks

**DOI:** 10.1186/1471-2105-7-280

**Published:** 2006-06-02

**Authors:** David J Reiss, Nitin S Baliga, Richard Bonneau

**Affiliations:** 1 Institute for Systems Biology, 1441 N. 34th St. Seattle, WA 98103-8904, USA; 2 New York University Dept. of Biology, Dept. of Computer Science, New York, USA

## Abstract

**Background:**

The learning of global genetic regulatory networks from expression data is a severely under-constrained problem that is aided by reducing the dimensionality of the search space by means of clustering genes into putatively *co-regulated *groups, as opposed to those that are simply *co-expressed*. Be cause genes may be co-regulated only across a subset of all observed experimental conditions, *biclustering *(clustering of genes *and *conditions) is more appropriate than standard clustering. Co-regulated genes are also often functionally (physically, spatially, genetically, and/or evolutionarily) associated, and such *a priori *known or pre-computed associations can provide support for appropriately grouping genes. One important association is the presence of one or more common cis-regulatory motifs. In organisms where these motifs are not known, their *de novo *detection, integrated into the clustering algorithm, can help to guide the process towards more biologically parsimonious solutions.

**Results:**

We have developed an algorithm, cMonkey, that detects putative co-regulated gene groupings by integrating the biclustering of gene expression data and various functional associations with the *de novo *detection of sequence motifs.

**Conclusion:**

We have applied this procedure to the archaeon *Halobacterium *NRC-1, as part of our efforts to decipher its regulatory network. In addition, we used cMonkey on public data for three organisms in the other two domains of life: *Helicobacter pylori, Saccharomyces cerevisiae*, and *Escherichia coli*. The biclusters detected by cMonkey both recapitulated known biology and enabled novel predictions (some for *Halobacterium *were subsequently confirmed in the laboratory). For example, it identified the *bacteriorhodopsin *regulon, assigned additional genes to this regulon with apparently unrelated function, and detected its known promoter motif. We have performed a thorough comparison of cMonkey results against other clustering methods, and find that cMonkey biclusters are more parsimonious with all available evidence for co-regulation.

## Background

The statistical elucidation of genetic regulatory networks from experimental data (commonly mRNA expression levels) is an important problem that has been the center of a large body of work [29,43]. Because this problem is *underconstrained *(the number of free parameters is far greater than the dimensionality of the data), many efforts include some means for dimensionality reduction. A common practice for reducing the dimensionality of this problem space has been to *cluster *genes into *co-expressed *groups based on their expression profiles, prior to network inference. Such a practice has the additional advantage that, if done properly, the signal-to-noise in the data can thereby be reduced through signal averaging. The genes in such clusters are often assumed to be *co-regulated, i.e*. to share the same regulatory controls, thereby implying biological relevance for such a pre-clustering step. However, gene transcript levels can be correlated either by chance (due to experimental noise or systematic error) or because of indirect effects, and therefore they might not actually be directly co-regulated. The integration of additional biologically-relevant evidence into a clustering procedure may be used to provide constraints on the identification of groups of co-regulated genes.

Co-regulated genes are often functionally (physically, spatially, genetically, and/or evolutionarily) linked [33,34,58,63,66,67]. For example, genes whose products form a protein complex are likely to be co-regulated. Other types of associations among genes, or their protein products, that (can) imply functional couplings include (a) presence of common cis-regulatory motifs; (b) co-occurrence in the same metabolic pathway (s); (c) cis-binding to common regulator(s); (d) physical interaction; (e) common ontology; (f) paired evolutionary conservation among many organisms; (g) common synthetic phenotypes upon joint deletion with a third gene; (h) sub-cellular co-location; and (i) proximity in the genome, or in bacteria and archaea, operon co-occurrence. These associations can be either derived experimentally or computationally (either pre-computed ahead-of-time, *e.g*. [23,60,62], or on-the-fly during the clustering process); indeed it is common practice to use one or more of these associations as a *post-facto *measure of the biological quality of a gene cluster. However, it is important to note that some of these data types, to varying degrees, can contain a high rate of false positives, or may imply relationships that have no implication for co-regulation. Therefore in their consideration as evidence for co-regulation, these different sources of evidence should be treated as priors, with appropriately different weights, based upon prior knowledge (or assumptions) of their quality and/or relevance.

Because a biological system's interaction with its environment is complex and gene regulation is multi-factorial, genes might not be co-regulated across all experimental conditions observed in any comprehensive set of transcript or protein levels. Also, genes can be involved in multiple different processes, depending upon the state of the organism during a given experiment. Therefore, a biologically-motivated clustering method should be able to detect patterns of co-expression across subsets of the observed experiments, and to place genes into multiple clusters. So-called *biclustering *(clustering both genes and experimental conditions), is a widely studied problem and many different approaches to it have been published [6,25,52,76,80,86,98]. Unlike standard clustering methods, most biclustering algorithms place genes into more than one cluster. Because biclustering is an NP-hard problem [25], no solution is guaranteed to find the optimal set of biclusters. However, many of these procedures have successfully demonstrated the value of biclustering when applied to real-world biological data (*e.g*. [6,56,88]).

We have previously described a procedure, the INFERELATOR [22], for learning global regulatory influences from expression data using continuous models of transcript levels. For this analysis (and most regulatory network inference algorithms), a pre-clustering step is desired to reduce the dimensionality of the data and enable noise reduction through signal averaging of clustered gene profiles. Low-level (but still significantly coherent) changes in expression of the clusters play an important role in constraining the model parameters, and the inclusion of these conditions in the biclusters can be important. Thus, a trade-off needs to be found between including as many experiments as possible in each cluster (to increase the constraints on the model parameters), while enforcing that these experiments be co-expressed. Different biclustering methods have different models of a "perfect" bicluster; for example constant rows/columns, coherent values, coherent "evolution" [56]. For our modeling purposes, only methods which derive biclusters with coherent, or correlated, gene profiles, such as those of Cheng and Church [25], Yang *et al*. [98], and Lazzeroni and Owen [53] are suitable. For example, algorithms which identify biclusters with constant levels of activation and/or repression [6,86] and/or which discretize the data [80] do not contain low or intermediate-levels of expression changes to constrain the regulatory network inference; indeed they often do not generate biclusters with many experimental conditions at all. Our analysis and previous reviews [6] of the Cheng and Church (CC) algorithm [25] show that it is not suitable for large-scale expression analysis. It, and the Plaid models of Lazzeroni and Owen [53] both produce biclusters that focus on low-variance sub-matrices of the expression data. The FLOC algorithm of Yang *et al*. [98] (an update to the CC algorithm which can handle missing values) provided the early inspiration for this work, which is essentially a re-formulation of their *δ*-cluster model with a basic probabilistic model for the expression data. This enables a more rigorous and intuitive integration of the model of expression data with models for the additional data types, as well as with prior distributions for constraining bicluster sizes and redundancy.

Guided by these motivations and requirements, we herein describe an algorithm that detects genes putatively co-regulated over subsets of experimental conditions by integrating the biclustering of gene expression data and multiple gene association networks with the *de novo *detection of cis-regulatory motifs. We applied this method to a global expression data set collected for the archaeon *Halobacterium *NRC-1, to find co-regulated gene sets as part of our ongoing efforts to model its regulatory network, and we present detailed evidence for the biological utility of this procedure as part of our modeling procedure. In addition, we used cMonkey to compute co-regulated gene clusters for three additional organisms in the two remaining domains of life: *Helicobacter pylori, Saccharomyces cerevisiae*, and *Escherichia coli*. The biclusters are presented to the biologist using the interactive visualization tools, the *Gaggle *[79] and *Cytoscape *[78], at our web site [4].

## Results

In this section we summarize the results of the application of our algorithm to four organisms, and describe its usefulness as a first step in our modeling of the *Halobacterium *regulatory network in conjunction with the Inferelator [22]. We perform a detailed analysis of its capabilities and assess its global performance, both internally and in comparison to other biclustering methods. The complete set of biclusters for all organisms are available for exploration using *Cytoscape *and the *Gaggle *[78,79] at our web site [4].

### The bacteriorhodopsin regulon in Halobacterium

**Figure 1:**
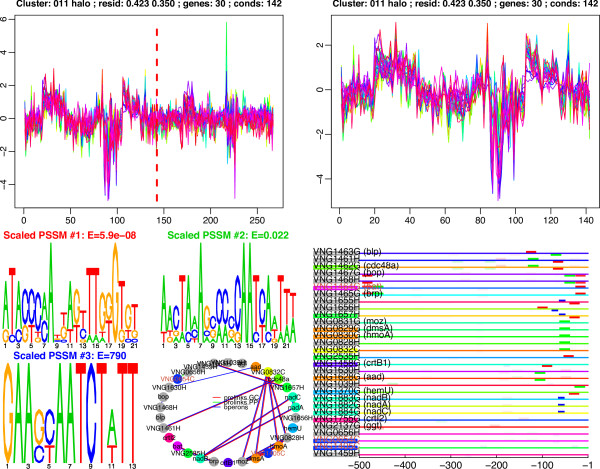
Bacteriorhodopsin *Halobacterium *bicluster with known Bat-binding motif (UAS). **A**: expression ratios of the bicluster's genes, over all experimental conditions (conditions within the bicluster are to the left of the red dotted line). **B**: expression ratios over only the conditions within the bicluster. **C**: motif logos [74] and *E*-values [10] for motifs that were detected in the bicluster. **D**: network of associations between the bicluster's genes in the various association networks used by CMONKEY, including operons, KEGG [48] metabolic pathways ("met" – see Methods; only present in Figures 4 and 5), and Prolinks [23] associations. The nodes are color coded by COG [89] functional groupings. Genes labeled in red text encode known or putative transcriptional regulators. **E**: diagram of the upstream positions of the motifs, colored red, green and blue for motifs #1, 2 and 3, respectively. The genes' names are color-coded by COG functional annotation as in the network subfigure. The colors of the lines for each gene's sequence correspond to those in the expression ratio plots.

**Figure 2:**
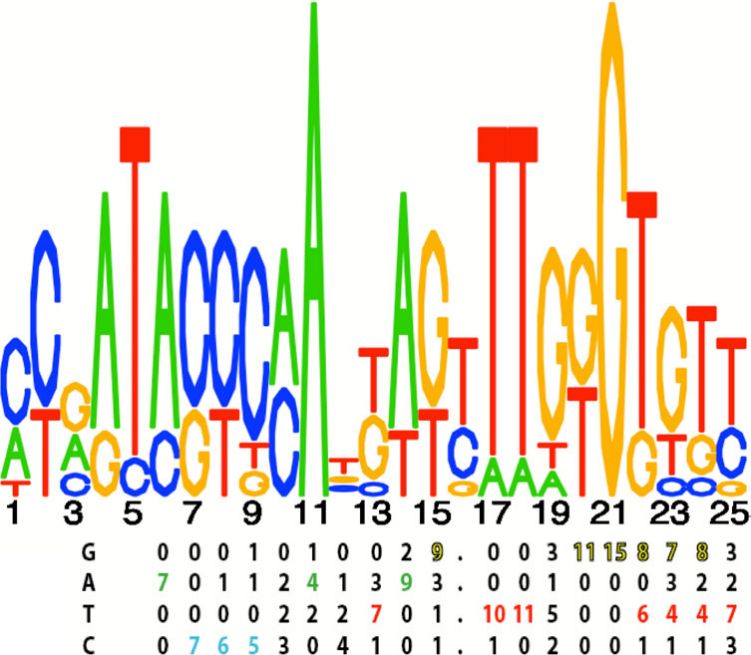
Motif logo for Bat-binding motif discovered in the bicluster of Figure 1 (top) compared to the saturation mutagenesis pattern observed for this regulator [12] (bottom).

The induction of phototrophic growth of *Halobacterium NRC-1 *under anaerobic conditions triggers the synthesis of bacteriorhodopsin (bR; a complex of the protein Bop and retinal), a light-driven proton pump that is further assembled into a purple membrane. Br is the major component of *Halobacterium *phototrophy, one of two anaerobic ATP generation pathways utilized by the organism [14]. Four genes responsible for bR synthesis (Bop and isoprenoid synthesis genes), *bop, brp, bat*, and *crtB1*, are co-regulated by Bat [13] through a common transcription factor motif that was characterized by saturation mutagenesis (the Bat UAS) [12]. This is the most well-studied regulon in *Halobacterium*, and the only one whose cis-regulatory motif has been experimentally verified. Bicluster #11 (Fig. 1) recapitulates much of what is known about this regulon, including all four bR genes, and a very close match to the Bat UAS (Figure 2). The additional genes in this bicluster are consistent with the co-regulation of bR with anaerobic respiration, including phytoene synthases, members of a DMSO-related operon [64], alcohol dehydrogenases, and an iron transporter. While the Bat UAS is not found upstream of many of these latter genes, a second significant motif (which was found upstream of the bR operon as well) was identified by cMonkey upstream of these genes.

Table 1 shows correlations between and among genes containing the putative Bat UAS (denoted "bR genes") and between and among genes containing the 2nd detected motif (denoted "DMSO genes"), over experiments within and outside the bicluster. While the bR genes are as tightly correlated with each other *outside *the bicluster as they are with the DMSO genes *inside *the bicluster, they are significantly less-correlated with the DMSO genes outside the bicluster (*p *¡ 0.026; paired *t*-test), and vice versa (*p *¡ 0.00095). This suggests that cMonkey partitioned the experiments between those in which the regulator which binds to the 2nd motif is controlling most of the genes in the bicluster (thereby causing them to appear tightly co-expressed) while over the conditions outside the bicluster, Bat is active, binds to the UAS, and bifurcates the regulation of the two sets of genes. Thus, cMonkey identified a novel relationship between phototrophy and DMSO (two of the four ATP-generating pathways available to *Halobacterium*), implying that the organism produces energy simultaneously via these two pathways under some environmental conditions.

The bicluster also includes *cdc48a*, which encodes a cell-division cycle – associated protein, with a strong match to the Bat UAS. We note that initial studies of the Bat UAS suggested that the regulatory sequences of as many as 108 genes contain instances of the motif [12]; clearly not all of these instances are active over the experiments used here. No similar bicluster, in terms of completeness of gene membership or similarity of motifs detected (via MEME [10]) to the Bat UAS, was found using other bi/clustering methods (see below for a list of methods attempted). When the cMonkey motif-detection component was turned off (see below), the UAS was not detected.

### SirR as a regulator of transport processes in Halobacterium

**Figure 3:**
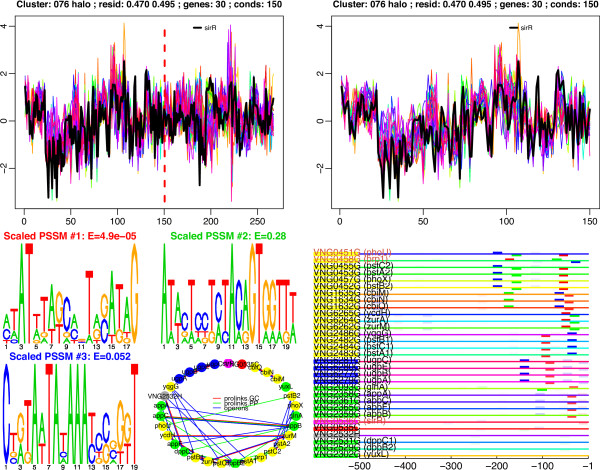
*Halobacterium *bicluster containing genes encoding the members of several transporter complexes. While *sirR *was not included by CMONKEY in the bicluster, we have added it to the figure and highlighted its expression profile.

cMonkey detected a bicluster (#76, Figure 3) primarily composed of transporter genes, including two phosphate transport systems, Co(II) transporters, a Mn(II) uptake system, glycerol phosphate transporters, and two peptide transport systems. While the phosphate, peptide, and Mn(II) transport systems might have been included in the bicluster by virtue of their functional associations, the glycerol phosphate and Co(II) transport system genes appear to have been included due to a strong match in the biclusters' putative motif #1. We can hypothesize that motif #1, which is present upstream to 24 out of the bicluster's 30 genes, is responsible for the high degree of expression correlation over ~150 conditions in this bicluster. None of the other bi/clustering methods tested identified a cluster containing the complete set of these transporters that enabled the generation of this type of model of the joint regulation of transporter activity in *Halobacterium*.

A potential advantage of the inclusion of *de novo *motif detection as part of the cMonkey biclustering procedure is that, for transcription factors that are not autoregulated, motif detection can break the causal symmetry between regulator targets and regulators controlling those targets. For example, an activator and several of its targets might seem co-expressed (and would therefore be placed in the same bicluster) when considering expression data alone. The absence of the regulator's binding site from its upstream sequence could, however, cause cMonkey to exclude the regulator from the bicluster, and thus assist any subsequent regulatory network inference on that bicluster. Although the above case is somewhat idealized, we find specific examples where motif detection correctly separates co-regulated groups from the co-expressed super sets that merge regulators and their targets together. SirR was predicted to regulate bicluster #76 [22] and this relationship was confirmed via a *sirR *knockout experiment [49]. SirR is annotated as an iron-dependent regulator in *Staphylococcus epidermis *and *Staphylococcus aureus *and is associated with Mn and Fe stress response in other microbial systems [44]. While *sirR *is correlated with the bicluster (Pearson correlation of 0.77, versus 0.69–0.92 for the genes in the bicluster), it was omitted from the bicluster by cMonkey, in part due to the poor match of *sirR's *upstream sequence to the bicluster's significant motif #1. While PhoU and Prpl (the transcriptional regulators that were included in bicluster #76) are also putative regulators of genes in bicluster 76, the inclusion of motif detection (along with the high stringency for co-expression used by cMonkey) suggests that SirR may have a more general role in the regulation of these transporter genes than PhoU and Prpl.

### Regulation of flagellar biosynthesis in E. coli and H. pylori

**Figure 4:**
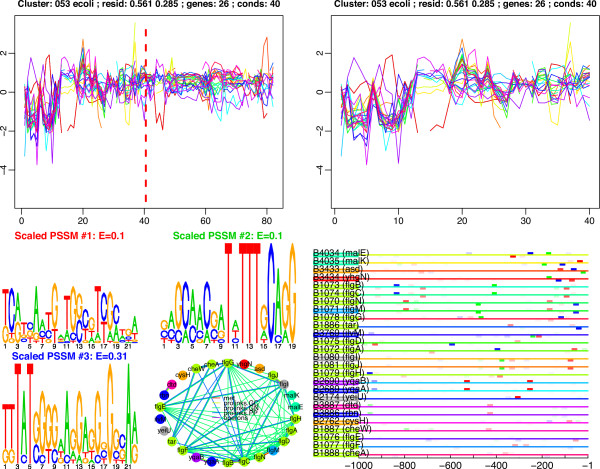
Flagellar biosynthesis bicluster from *E. coli*. Motifs #1 and 2 make up part of the *σ*^70^(RpoD)/FlhDC activator complex binding site for activation of Class-2 flagellar genes.

In *E. coli*, the repertoire of more than 50 genes that encode proteins involved in motility (flagellar and chemotaxis system) are regulated in a cascade that can be separated into three classes. These regulatory classes correspond to the ordering of the genes' temporal requirement during flagellar assembly [7,26,47]. Class-2 genes are regulated by an RpoD/*σ*^70 ^and FlhDC activation complex, and encode flagellar structural and assembly proteins and two regulators (*fliA *and *flgM*)*. fliA *and *flgM *subsequently activate the Class-3 operons (which include chemotaxis signaling and flagellar activation/motion-associated genes) [26]. cMonkey detected a bicluster in *E. coli *(Fig. 4) that is enriched in flagellar biosynthesis genes (including the regulator *flgM*)*; *most of these genes' upstream sequences contain motifs (#1 and #2 in Fig. 4) that correspond to the known promoter binding site for this activator complex [26]. While several other bi/clustering methods (see below for details), such as *k*-means and SAMBA, detected clusters that were enriched in both flagellar- and chemotaxis-associated genes, we were unable to detect the *σ*^70^/FlhDC binding motif in any of these clusters due to the presence of many additional unrelated sequences that added noise to the search. The cMonkey bicluster included only two (of 11) annotated "chemotaxis"-related genes (which are all in Class-3, and do not contain the detected motif), whereas the larger SAMBA bicluster, for example, did not discriminate between these two related functions (containing 9 of the 11 genes). If MEME [10] is run independently on upstream sequences of the flagellar function-annotated genes (43 in all), it detects the *σ*^70^/FlhDC binding motif in ~20 of them, while it does not detect a motif for the 11 chemotaxis-annotated genes (nor in the combined set of 54 sequences). This analysis suggests that while many genes in both Class-2 and Class-3 are co-expressed in the *E. coli *data, cMonkey can correctly separate the two classes on the basis of motif detection and association networks.

**Figure 5:**
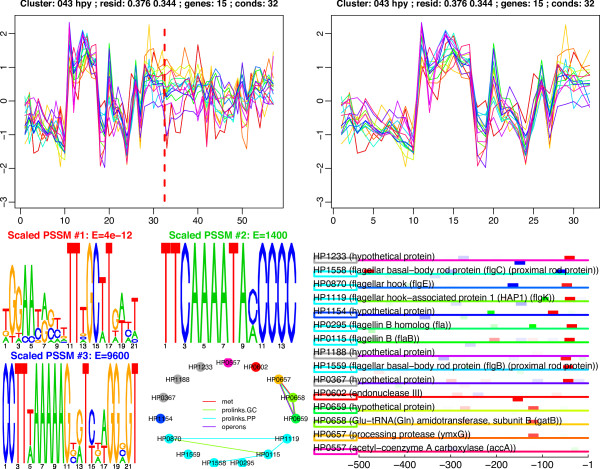
Flagellar-function *H. pylori *bicluster with known RpoN-binding motif (motif #1).

The *H. pylori *cluster in Fig. 5 is also highly enriched in Class-2 flagellar-associated genes, many of which are associated with the RpoN/*σ*^54^-regulated flagellar regulon [65]. The most significant motif detected in this cluster corresponds to the RpoN binding site: 5'-GGaa-N5-tttGCtT-3' [65] that is similar to the *σ*^70 ^binding site in *E. coli *[26]. Other biclustering algorithms identified biclusters in the *H. pylori *data containing some of the same genes as this cMonkey bicluster, however most of those clusters contain > 50 additional genes (several with > 200), and thus the RpoN-binding motif was undetectable for clusters generated by any of these methods. Individual clusters found using hierarchical clustering (*k = *300) and fc-means (*k = *50) on the *H. pylori *data had matches to this motif, suggesting that because the data set is small (~60 experiments), biclustering is not always necessary here. However, neither of these respective clusters were as complete in their list of genes with the RpoN-binding motif as was the cMonkey version (6 of 6 for the hierarchical clustering cluster, and 12 of 19 for the *k*-means cluster, versus 14 of 15 for cMonkey). The similarity in function and putative regulatory motifs for these two orthologous biclusters points to the potential future use of algorithms such as cMonkey for cross-species analyses of gene regulation [46,85].

### A novel putative ricin-like toxin in H. pylori

The integrated analysis of the full set of biclusters in the context of additional biological knowledge (such as detailed annotations for individual genes) can result in biological insights into the combined roles of multiple biological modules. Such an analysis requires the presentation and integration of cMonkey biclusters with the visualization and exploration tools *Cytoscape *[78] and the *Gaggle *[79] (see below for details). An illustrative example in *H. pylori *involves a group of biclusters containing CAG pathogenicity genes. It has been hypothesized that a drop in pH may act as a signal to induce genes encoding several virulence factors including CagA (Cag26), which upon injection into target cells plays a role in the early events of gastric colonization. A known promoter motif TTTTAA [61,94] appears conserved upstream to several of these pH-induced genes. Several biclusters were detected which contain this motif and numerous pathogenicity island genes, including *cag8, cag12*, and *virB11*, which encode type IV secretion system proteins and *flaA *and *flaB*, which encode key flagellin subunits [32]. Other processes represented in these biclusters include outer membrane biogenesis (*omp*5, *omp9*, *omp29*) and peptidoglycan biosynthesis (*murC, murF and murG*)- which have all been implicated as important for pathogenesis [81,95]. Through the analysis of these related biclusters and their common motif, we identified a novel putative ricin-like toxin among the un-annotated *H. pylori *genes (HP1028) [79].

### Biclusters in S. cerevisiae

The algorithm detected many strongly significant biclusters in *S. cerevisiae*, many of which with known or previously-observed cis-regulatory motifs, and combinations thereof. Some examples of these are included in [Additional File 1]; all cMonkey-generated yeast biclusters may be viewed and explored using *Cytoscape *and the *Gaggle *[78,79] at our web site [4]. Histograms of the positions of the detected motifs in the yeast upstream sequences show a marked peak near -150 bp, which hints that many of the motifs identified by cMonkey for *S. cerevisiae *are functional, since the motifs are actually searched for in the first 500 bp upstream of each gene [see Additional File 1, Figure Twelve].

### Validation and comparisons with available methods

#### Tracking the cMonkey optimization

**Figure 6:**
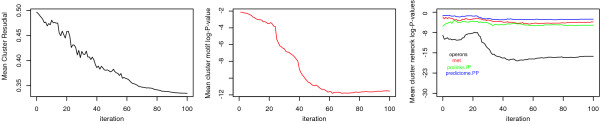
Mean external measures of *Halobacterium *bicluster "quality", as a function of iteration of bicluster optimization. Left: co-expression ("residual," [98]). Center: motif co-occurrence ("Motif log(*p*-value)"). Right: mutual clustering coefficient (log-*p*-value [37]) in four different association networks: operons, KEGG [48] metabolic pathways ("met" – see Methods), and Prolinks [23] associations.

By tracking the mean progression of all biclusters during their optimization, we can quantify the degree to which the biclusters improved with regard to each model component (data type). Examples of such measures for *Halobacterium *are shown in Fig. 6. The scores shown are mean bicluster residual [98], the mean motif log-p-value [10], and mean log *p*-values of mutual clustering coefficient in certain association networks [37]. It is clear that most of these measures greatly improve (*i.e*. decrease) throughout the optimization, even though the procedure is *not *optimizing any of the "scores" that are plotted in Fig. 6; rather it is optimizing a joint discriminative model that includes terms which are related to these measures. We obtain similar trends in cMonkey runs on all organisms [see Additional File 1, Figure Ten].

#### Testing the cMonkey model

##### Tests of data integration

We tested whether cMonkey is correctly optimizing the joint model with respect to the different data types by varying the weights which parameterize the influence of each of those data types on the joint model (the default for these mixing parameters is set such that the three major data types have roughly equivalent influence). When we down-weight the mixture parameter for a given data type and thus eliminate its influence on the bicluster optimization, as expected, we find that this down-weighted component is poorly-optimized. At the same time, the remaining components are almost always optimized better. Thus each model component serves to regularize the bicluster model, preventing the biclusters from being over-fit to one or more individual subsets of the data. Not surprisingly, we also find that when certain components are up-weighted, they are better optimized, at the expense of a somewhat diminished ability to optimize the remaining components. [Additional File 1, Figure Fifteen] displays mean measures of bicluster quality (here, residual against motif log-*p*-value) for these different cMonkey runs with weights adjusted in this manner (here, on the *S. cerevisiae *data). These tests show that our inclusion of the three data types results in biclusters that simultaneously satisfy our joint model better than biclusters supported by subsets of the data types (model components). A similar conclusion may be drawn from comparisons of these different cMonkey runs to "external" sources of evidence (see below and [Additional File 1, Figures Sixteen and Eighteen]).

##### Additional tests of the relationship between multiple data types and model components

By successively removing individual components of the model, we can also characterize relationships that exist between an individual data type and the others, that have not been removed, by observing the degree to which the optimization of the removed data type still improves. For example, by turning off an individual network *N *(setting q0N
 MathType@MTEF@5@5@+=feaafiart1ev1aaatCvAUfKttLearuWrP9MDH5MBPbIqV92AaeXatLxBI9gBaebbnrfifHhDYfgasaacH8akY=wiFfYdH8Gipec8Eeeu0xXdbba9frFj0=OqFfea0dXdd9vqai=hGuQ8kuc9pgc9s8qqaq=dirpe0xb9q8qiLsFr0=vr0=vr0dc8meaabaqaciaacaGaaeqabaqabeGadaaakeaacqWGXbqCdaqhaaWcbaGaeGimaadabaGaemOta4eaaaaa@3057@ to zero), we can rank that network with respect to the degree to which it improves (using the scores described above) when the other components (co-expression, motifs, and other networks) are optimized. For example, we find that the operon associations and protein-DNA interaction networks are well-optimized via the indirect optimization of co-expression, while metabolic pathways and phylogenetic profile associations show weaker, but still significant, correlation to co-expression. Protein interaction networks and Rosetta Stone associations appear to be the least-significantly correlated with co-expression, possibly due to their higher false-positive rate. Carrying out this type of analysis on-the-fly could allow us to iteratively update the weighting parameters as cMonkey optimizes the biclusters (so-called "Pareto-front" optimization [93]).

##### Randomization and shuffling tests

As an alternative to the difficult task of generating biologically realistic "synthetic" data, we chose to randomize the data instead, in order to further assess the significance of patterns discovered by cMonkey. If we completely shuffle an individual data type, then we effectively eliminate any signal that exists in that component but preserve any influence that the noise component of that data type adds to the procedure (possibly interfering with optimization of other model components). The resulting effect is very similar to strongly down-weighting that component of the model, as described above. A more stringent test can be performed by randomizing only the associations between each gene's expression data, its sequence, and its location in the association networks. This preserves the higher-order structure of each data type, but scrambles the mutual support each data type might present to the overall model. On data randomized in this manner, cMonkey is unable to find biclusters that, on average, are as well-optimized (in terms of the "scores" described above) as in the original data. The significance of this result varies depending upon the organism and the quality and amount of data available; on the *Halobacterium *data, this type of data shuffling results in average bicluster residuals ~20% higher, and average motif *p*-values ~1 log_10_-unit higher than in the un-shuffled data. The algorithm does not find significant association subnetworks in any of the shuffled trial runs.

#### Comparison of cMonkey with other methods

In our assessment of cMonkey's performance, we compared cMonkey-generated biclusters against those generated using the following algorithms: Cheng-Church (CC [25]), Order Preserving Sub-matrix (OPSM [18]), Iterative Signature (ISA [19]), xMOTIF [55], BIMAX [6], and SAMBA [86]. We also compared our method to hierarchical clustering and *k*-means clustering [30] with *k *varying between 10 and 300 (see Methods for details). In addition, we performed these analyses on cMonkey runs with various model parameters up- and down-weighted, as described above, to demonstrate the effect of including various subsets of the cMonkey model components in the comparisons. Additional details on the analysis are provided in the Methods section; supporting figures are shown in [Additional File 1]. All bi/clusters generated by the various algorithms are available for interactive exploration via *Cytoscape *and the *Gaggle *[78,79] at our web site [4].

##### Comparison in the context of regulatory network inference

A major motivation of this work is to provide a method for deriving co-regulated groups of genes for use in subsequent regulatory network inference procedures. To do this, we wish to find coherent groups of genes over those conditions with a large amount of variation. In other words, we are hoping to detect submatrices in the expression data matrix which are coherent and simultaneously have high information content or overall variance. In addition, we need to find biclusters with many conditions/observations included, as this increases the significance of each bicluster and also of the subsequently inferred regulatory influences for that bicluster. Some relevant summary statistics of the runs of various algorithms on all four organisms are listed in [Additional File 1, Table Two]. In general we see that cMonkey generates biclusters with a significantly greater number of experiments than the other methods. Even with this additional constraint (*i.e*. including a greater number of experiments in the clusters) and further constraints that cMonkey imposes with the association- and motif- priors, the algorithm in general generates biclusters with a "tighter" profile, as measured by mean bicluster residual [25]. Thus, we find that biclusters generated by cMonkey are generally better-suited for inference algorithms such as the Inferelator [22], and potentially other linear or continuous models as well. We tested this by running the Inferelator on biclusters generated by SAMBA [86] for *Halobacterium *and then comparing the predictive performance of the resultant regulatory network models on newly-collected data, relative to those generated for cMonkey-generated biclusters [22]. We found that, largely due to the smaller number of experiments included in SAMBA biclusters, the inferred network was significantly less able to predict new experiments (an increase in the predictive error from 0.368 to 0.470; *p*-value of difference by *t*-test = 1.0 × 10^-22^).

##### Comparison against external measures

Defining an unbiased external measure of "success" of a bi/clustering algorithm is a very difficult problem [30]. In fact, even if a good, unbiased measure were to be found, a comparison of different bi/clustering results in the context of that measure is also not straightforward. We have attempted to estimate various measures of success of different algorithms in various contexts, with regard to sensitivity, selectivity, and two measures of coverage, in order to provide the reader with a fair comparison of cMonkey with other previously published methods. We define the *sensitivity *of a bi/cluster set as the commonly-used fraction of bi/clusters that are significantly enriched with genes that (a) have the same functional annotation in GO [40] or KEGG [48], or (b) contain a known cis-regulatory motif [60], or (c) mimic groups of co-regulated genes, from experiments such as ChlP-chip assays [39]. These measures are shown for *S. cerevisiae *[Additional File 1, Figure Sixteen (A-D)] and for *Halobacterium *[Additional File 1, Figure Eighteen (A-D)] for the different algorithms. Bi/cluster *specificity *measures how well the bi/clusters segregate genes along the same lines as the different Classes; here, we use a measure of the fraction of genes in each significantly-annotated bi/cluster that have the same significantly-enriched annotation(s) found for that bi/cluster. We use *coverage *to describe two distinct measures: (a) the fraction of all observed genes and experimental conditions in the data which are included in at least one bi/cluster [Additional File 1, Table Two], and (b) the fraction of all groups in a given Class that are significantly enriched in at least one bi/cluster for *S. cerevisiae *[Additional File 1, Figure Sixteen E] and for *Halobacterium *[Additional File 1, Figure Eighteen E]. We should note that it is debatable which of these metrics of bicluster quality represent the best measures of "correctness" for a bi/clustering method. For example, genes that modulate the protein and transcript levels of other proteins might have similar GO functional categories (protein degradation, transcription factor, regulation, etc.) but may be correctly partitioned separately with the processes they individually regulate. It is also important to note that all of these statistical measures of bi/cluster validity contain inherent flaws or biases that correlate strongly with bi/cluster size, overlap degree, and gene coverage. For example, OPSM generated 8 biclusters which excluded less than ~1/2 of all measured genes from its clusters, yet it outperforms all other methods in the sensitivity measure. We have used the false discovery rate (which is larger for bigger clusters) to correct these *p*-values for multiple testing (see Methods), however, we still find a size bias in the corrected scores (which is also seen in previously-written comparisons of biclustering methods, *e.g*. [6]). In addition to GO and KEGG, we assess bi/clusters against known cis-regulatory motifs [39,60], and high-throughput protein-DNA interaction sets [39]. We included the runs from various test parameterizations of cMonkey in the analysis (see above), so the effect of the different input data sets could be seen. We also divided each tested bicluster set into "BIG" and "SMALL" halves, so that the size-related biases in this measurement may be seen and accounted for in the comparisons (for example, the BIG half of cMonkey's bicluster set have about the same mean number of genes per bicluster as the SMALL half of SAMBA's bicluster set, which therefore makes them more readily comparable [see Additional File 1, Figures Seventeen and Nineteen]).

In general, we find that cMonkey performs well in comparison to all other methods when the trade off between sensitivity, specificity, and coverage is considered, particularly in context of the other bulk characteristics (cluster size, residual, etc.). We find that SAMBA also performs well when these measures are considered; however because its biclusters contain on average 3 × more genes than cMonkey's, and far fewer experiments (and therefore SAMBA, like most other methods, cover less of the data space), the direct comparison is difficult. cMonkey, as it was designed to do, covers more of the data space (and therefore more of the different Classes defined above) for each organism, and it is therefore more suitable for our regulatory network learning motivations. In particular, while it includes far more experiments per cluster and restricts its clusters to have significantly tighter co-expression, it still does comparably well when assessed against the external measures due to its data integration. [Additional File 1, Figure Sixteen] shows, for example, that the cMonkey runs carried out with the association networks up-weighted, in particular, do partition the functional classes better (and vice versa when they are turned down). The final judgement is that because cMonkey biclusters do a better job at regenerating the expression data than other methods, and at least a comparable job at recapitulating the external (as well as internal) measures of bicluster quality, they are, overall, more parsimonious with, and more generative of the patterns found in the available data. Thus, cMonkey biclusters are arguably well-suited for the inference of gene co-regulation and regulatory networks, in comparison to available bi/clustering methods.

### Bicluster visualization

**Figure 7:**
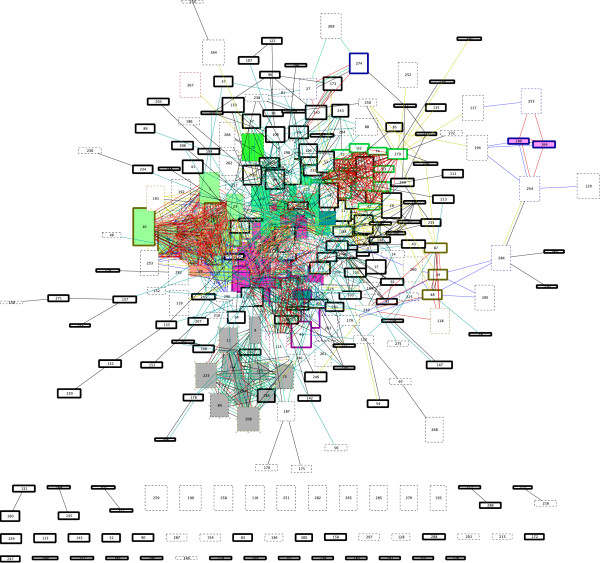
*Halobacterium *bicluster network as visualized using *Cytoscape *[78]. Biclusters are represented as rectangular nodes, colored based upon significant functional annotations [40]. Different colored edges represent different measures of cluster similarity or connectivity in various association networks (dark blue: KEGG [48] metabolic pathways; dark red: GO [40] functional similarity; light blue: motif similarity; yellow: operon membership; light red: COG [89] functional similarity; green: gene membership). Highly-connected (and therefore functionally-related) biclusters are placed near each other in the layout. The selected (grey) bicluster group near the bottom contains bacteriorhodopsin-associated biclusters, including the one in Fig. 1. Note that these biclusters have not been filtered to remove redundancy.

Because a population of biclusters will contain some overlapping elements which can confuse their interpretation, it is important to present them to the biologist in a format that promotes their interpretation and exploration in the context of supporting information, cMonkey automatically generates, for each bicluster, a "bicluster diagram" (example in Figure 5), presents to the biologist the bicluster's co-expression pattern, motif logos [74] and upstream sequence locations (in this study, for as many as three detected motifs), as well as the various functional associations among the bicluster's gene members. We have found that a useful and intuitive visualization scheme for a population of overlapping and often redundant biclusters is via an association network (Figure 7) of rectangular bicluster nodes (whose sizes are proportional to their gene/condition membership); analogous to "module networks" published in previous works. We visualize this bicluster network using *Cytoscape *[78]. Each bicluster is annotated with its gene and condition members, a measure of its co-expression, significant functional annotations (GO [40], KEGG [48] and COG [89]), and significant motifs. Edges are drawn between two biclusters if they contain non-redundant genes which are connected individually in any association networks. Connections are also added between pairs of biclusters that have a large amount of overlap in gene membership, motif similarity, expression correlation, and/or functional annotation. A spring-embedded layout algorithm [83] is used to spatially organize the network, placing highly-connected (and therefore related) biclusters spatially closer to each other. As a result, groups of biclusters with common function(s), or which lie in adjacent biochemical pathways, may be easily identified in the network, as shown in Figure 7. The integration of *Cytoscape *with the *Gaggle *[79] automatically cross-references biclusters with their respective "bicluster diagrams", and enables searching and browsing of additional biological information (such as expression data submatrices, gene browsers, annotation databases) or further analysis (*e.g*. via direct connection to *R*) of a bicluster's gene members, greatly facilitating their analysis.

## Discussion and conclusion

The integration of clustering or biclustering of expression data with additional information is a problem of growing interest. The method presented here may be compared favorably with several recently published clustering and biclustering algorithms that have integrated different types of data, including *de novo *detection of sequence motifs [75], known sequence motifs [28,54], and various types of association information [28,86,87]. We have (to date) seen each of these other methods applied primarily to yeast, which is unique in the quantity of data available relative to the complexity of its genome. Many aspects of our method are inspired by these works. cMonkey does not require discretization of expression data, and is therefore capable of capturing patterns in low-level responses, while still being robust to noise due to its integration of different types of biological information. For example, although the *H. pylori *and *E. coli *data was limited in size and quality (with many expression experiments containing only one replicate, and many missing values), we were able to detect several interesting biclusters with significant putative (or known) motifs. In addition, cMonkey includes a greater number of experiments in each bicluster than other methods, while still obtaining a higher amount of correlation among its gene members. Finally, cMonkey is model-based and variables (such as the distribution of bicluster sizes, and the distribution of overlap between biclusters) are parameterized using simple statistical distributions. Therefore, their adjustment is intuitive and understandable, as well as robust to varying data size and quality. In our experience, this is in contrast to other biclustering algorithms, which often require tweaking of *p*-value cutoffs, dimensionless variables, or thresholds, which often result in unpredictable effects on the biclusters' properties.

We believe that the ability for the cMonkey user to explicitly control the contribution of different data types through their weights opens up many potential uses for the algorithm beyond the basic identification of co-expressed clusters of genes. This flexibility enables the detection of biclusters which stress certain type(s) of biological information over others. Indeed, in many cases it is still not known whether a certain type of pair-wise association between genes is actually correlated with co-expression. Such "guilt-by-association" is often assumed, *e.g*. between co-expression and functional categories [97], but such conclusions can be controversial [11], as bioinformatics has "only codified a small proportion of the biological knowledge required to understand microarray data" [27] (obviously other types of associations, such as operon [69] or cis-motif co-occurrence are more strongly tied to co-expression). cMonkey users can easily choose to generate tightly-co-expressed biclusters that are strongly supported by evidence provided by one or more other sources of information for their system of interest, and they can do so by including them as highly-weighted components of the bicluster model. For example, they could (a) identify active or co-expressed signaling or regulatory pathways or complexes, as in [45], by up-weighting protein interaction networks or metabolic networks; (b) reconstruct metabolic pathways, by up-weighting the metabolic network and expression data, as in [50]; (c) attempt putative *de novo *cis-regulatory motif detection in newly-sequenced genomes (without expression data), by setting the expression weight to zero; (d) assess the quality of complete networks or individual edges in operon associations or protein-DNA interactions, as in [69], by up-weighting these associations and the expression data. Future improvements to the method could be made to learn the appropriate weights for each data type, from the data (rather than as input parameters), for example by using an unconstrained multi-parametric logistic regression as briefly described in the Methods section, or by adaptively constraining the weights such that no component of the model over-regularizes with respect to the other components (*e.g*. "Pareto-front" optimization [93]).

For sake of simplicity, flexibility and statistical transparency, we have used simple models for each of the individual data types and logistic regression to integrate them into a joint model. However, this simplicity comes at the expense of several trade-offs, which could be improved upon. Whereas it may be more appropriate to treat some associations as a property of sets rather than networks, we have treated all the same. Certain types of associations (such as protein-DNA networks and functional annotation classes) could be treated differently. In addition, any confidence values associated with individual edges in some of the networks are currently ignored. While edge weights could currently be included, for example, by dividing the high and low confidence edges into separate networks with different weights, it would be preferable to more cleanly model such association evidence. Third, we have reason to believe that our use of MEME for motif detection may be increasing our sensitivity to noise. The method could benefit from an assessment of different algorithms for detecting motifs in conjunction with biclustering, or the consensus of more than one method can be integrated, as in [39]. Also, as we move to more complex organisms, we find that multiple motifs cooperate in their regulation function, with conserved patterns, orientations, and upstream locations, such additional motif correlation and positional information may be exploited, with little modification to the current framework, to increase the sensitivity and specificity of identified motif patterns, such as via meta-MEME, [38] or others [20,68]. Also possible is the move toward the integrated multi-species biclustering of expression data, merging the multi-species clustering motivations of [83] with additional phylogenetic associations and motif detection (as in [96]).

Because the goals of the development of cMonkey are unique relative to previous biclustering methods (*i.e*. coupled to a continuous regulatory network inference procedure, such as the Inferelator [22]), the resulting biclusters have unique characteristics when compared to many previously-published methods. We have shown that the procedure "works harder" to insure that a greater percentage of genes that are observed in the data set are included in at least one cluster, while reducing redundancy between overlapping biclusters and maximizing the number of experiments that are included in each bicluster. Because of these characteristics, standard methods of assessment of biological relevance of cMonkey-generated clusters (*e.g*. by functional annotation over-representation) are far from ideal, as they do not account for varying bicluster sizes, redundancy, and coverage of the data. Choosing the appropriate biclustering procedure for one's needs therefore involves finding a balance of these different bicluster-set properties that returns the desired outcome. As was written by Patrick D'haeseleer, [30] "There is no one-size-fits-all solution to clustering, or even a consensus of what a 'good' clustering should look like."

## Methods

### Materials and data

#### Expression data

Expression data for *Halobacterium *were collected by members of the Baliga lab, containing genome-wide measurements of mRNA expression in 292 conditions, as described in full in [22] and references therein. Expression data for *H. pylori *and *S. cerevisiae *were collected from the Stanford Microarray Database [3]. Certain experiments such as strain comparisons, genomic DNA, and RNA decay experiments which are unlikely to relate to gene regulation were removed from the sets prior to analysis. This filtering resulted in 58 of an original 250 conditions for *H. pylori *and 667 of 1051 conditions for *S. cerevisiae*. Data for *E. coli *was compiled from publicly-available data provided to us by E. A1m, including 86 conditions. As a pre-processing step, genes were removed from the expression data for which there was not significant (1.5-fold) expression in any of the experiments. The data were then row-normalized (each gene's expression levels normalized to mean = 0, SD = 1). No further pre-processing or filtering of the microarray data was performed.

#### Association and metabolic networks

Genetic associations derived from comparative genomics, such as phylogenetic profile, Rosetta Stone, gene neighbor and gene cluster, were compiled from *Prolinks *[23] and *Predictome *[62] for all organisms. These networks include predicted operon "associations," which were also used to identify "unique" regulatory sequences that are to be used in the motif detection. Metabolic network reconstructions from the Kyoto Encyclopedia of Genes and Genomes [48] were represented as associations between two genes if they participate in a reaction sharing one or more ligands, after removing the most highly-connected ligands, such as water and ATP [16].

#### Interaction networks

*H. pylori *protein-protein interactions were collected from the global experiments of [70]. *S. Cerevisiae *protein-protein, protein-DNA, and genetic interactions were collected from DIP [73] and BIND [9]. The protein-DNA interactions were converted into a network of associations between all pairs of genes whose upstream sequences were found to bind to the same regulator(s).

#### Upstream sequences

Upstream sequences for all organisms were obtained from GenBank using the Regulatory Sequence Analysis Tools (RSAT [92]). Using these tools, we extracted 1000-bp cis-regulatory sequences. For bacteria and archaea, these sequences were shortened to 500 bp, and then "operon-shifted" using the *gene cluster *(operon association) networks from *Prolinks *[23] and *Predictome *[62]. Upstream sequences for genes in the same operon were converted to "operon-shifted" sequences by using the (same) upstream sequence of the first gene in the operon Similar "operon-shifted" upstream sequences were identified using BLASTN [8] using a 50 bp non-gapped alignment window, to avoid using multiple copies of the same sequence in the motif detection.

#### Functional annotations for comparison tests

Gene ontology (GO) [40] annotations for each organism were obtained from the European Bioinformatics Institute [1] and matched to annotation names obtained from the GO web site. KEGG annotations were downloaded from their web site [2]. Predicted and experimentally-derived DNA binding motifs were obtained for *S. cerevisiae *from [39,60], and for *E. coli *from [71,72]. When these binding motifs were provided as position weight matrices (PWMs), they were converted into regular expressions, in order to enable rapid scanning of upstream sequences.

### The bicluster model

#### Model overview

Each bicluster is modeled via a Markov chain process, in which the bicluster is iteratively optimized, and its state is updated based upon conditional probability distributions computed using the cluster's previous state. This enables us to define probabilities that each gene or condition belongs in the bicluster, *conditioned upon *the current state of the bicluster, as opposed to requiring us to build a complete (joint) model for the bicluster, *a priori*. The components of this conditional probability are modeled independently (one for each of the different types of information which we are integrating) as *p*-values based upon individual data likelihoods, which are then combined into a regression model to derive the full conditional probability. In this work, three major distinct data types are used (gene expression, upstream sequences, and association networks), and accordingly *p*-values for three such model components are computed: the *expression *component, the *sequence *component, and the *network *component.

**Figure 8:**
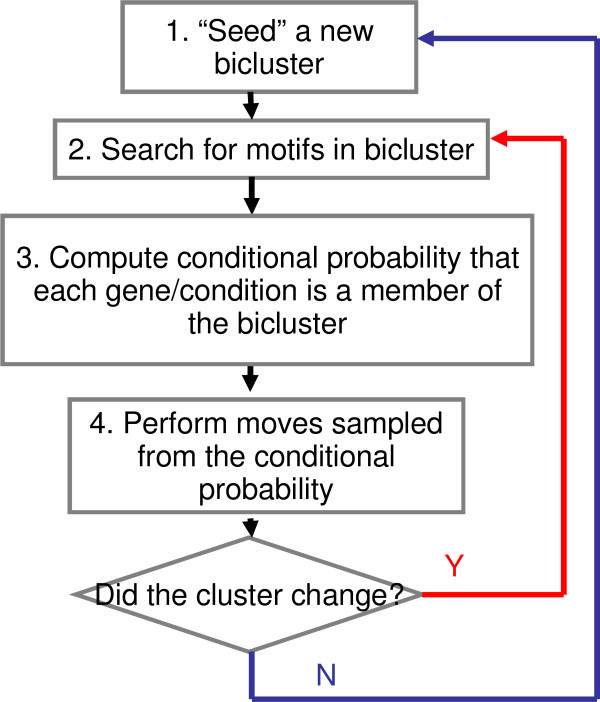
A schematic diagram of the CMONKEY biclustering procedure. The inner (red) loop depicts the optimization for each newly-seeded bicluster.

Each bicluster begins as a *seed*, or starting cluster, that is iteratively optimized by adding/removing genes and conditions to/from the cluster by sampling from the conditional probability distribution using a Monte Carlo procedure, to prevent premature convergence. Such an iterative machine learning technique is akin to a Markov chain Monte Carlo (MCMC) process. Additional clusters are seeded and optimized until a given number (*k*_max_) of clusters have been generated, or significant optimization is no longer possible. The complete process is shown schematically in Fig. 8, and described in detail below.

In the following discussion, let *i *be an arbitrary gene and *j *an arbitrary experimental condition. A bicluster *k *∈ **K **is fully defined by its set of genes **I**_*k *_and experimental conditions **J**_*k*_. The membership *y*_*lk *_∈ {0, 1} of an arbitrary gene *or *condition *l *in bicluster *k *is an independent Bernoulli indicator variable with conditional probability *p*(*y*_*lk *_= 1).

#### The expression component

The expression data is a set of measurements of genes *i *∈ **I **over experiments *j *∈ **J**, comprising a |**I**| × |**J**| matrix *x*_*ij *_∈ **X**. Each bicluster *k *defines a |**I**_*k*_| × |**J**_*k*_| submatrix *x*_*i'j' *_∈ **X**_*k*_: *i' *∈ **I**_*k *_⊂ **I**; *j' *∈ **J**_*k *_⊂ **J**. The variance in the measured levels of condition *j *is σj2=|I|−1∑i∈I(xij−x¯j)2
 MathType@MTEF@5@5@+=feaafiart1ev1aaatCvAUfKttLearuWrP9MDH5MBPbIqV92AaeXatLxBI9gBaebbnrfifHhDYfgasaacH8akY=wiFfYdH8Gipec8Eeeu0xXdbba9frFj0=OqFfea0dXdd9vqai=hGuQ8kuc9pgc9s8qqaq=dirpe0xb9q8qiLsFr0=vr0=vr0dc8meaabaqaciaacaGaaeqabaqabeGadaaakeaaiiGacqWFdpWCdaqhaaWcbaGaemOAaOgabaGaeGOmaidaaOGaeyypa0JaeiiFaWhcbeGae4xsaKKaeiiFaW3aaWbaaSqabeaacqGHsislcqaIXaqmaaGcdaaeqaqaaiabcIcaOiabdIha4naaBaaaleaacqWGPbqAcqWGQbGAaeqaaOGaeyOeI0IafmiEaGNbaebadaWgaaWcbaGaemOAaOgabeaakiabcMcaPmaaCaaaleqabaGaeGOmaidaaaqaaiabdMgaPjabgIGiolab+Leajbqab0GaeyyeIuoaaaa@494C@, where x¯j=∑i∈Ixij/|I|
 MathType@MTEF@5@5@+=feaafiart1ev1aaatCvAUfKttLearuWrP9MDH5MBPbIqV92AaeXatLxBI9gBaebbnrfifHhDYfgasaacH8akY=wiFfYdH8Gipec8Eeeu0xXdbba9frFj0=OqFfea0dXdd9vqai=hGuQ8kuc9pgc9s8qqaq=dirpe0xb9q8qiLsFr0=vr0=vr0dc8meaabaqaciaacaGaaeqabaqabeGadaaakeaacuWG4baEgaqeamaaBaaaleaacqWGQbGAaeqaaOGaeyypa0ZaaabeaeaacqWG4baEdaWgaaWcbaGaemyAaKMaemOAaOgabeaakiabc+caViabcYha8Hqabiab=LeajjabcYha8bWcbaGaemyAaKMaeyicI4Sae8xsaKeabeqdcqGHris5aaaa@401D@. We compute the mean expression level of condition *j *over the cluster's genes **I**_*k*_, x¯jk=∑i∈Ikxij/|Ik|
 MathType@MTEF@5@5@+=feaafiart1ev1aaatCvAUfKttLearuWrP9MDH5MBPbIqV92AaeXatLxBI9gBaebbnrfifHhDYfgasaacH8akY=wiFfYdH8Gipec8Eeeu0xXdbba9frFj0=OqFfea0dXdd9vqai=hGuQ8kuc9pgc9s8qqaq=dirpe0xb9q8qiLsFr0=vr0=vr0dc8meaabaqaciaacaGaaeqabaqabeGadaaakeaacuWG4baEgaqeamaaBaaaleaacqWGQbGAcqWGRbWAaeqaaOGaeyypa0ZaaabeaeaacqWG4baEdaWgaaWcbaGaemyAaKMaemOAaOgabeaakiabc+caViabcYha8Hqabiab=LeajnaaBaaaleaacqWGRbWAaeqaaOGaeiiFaWhaleaacqWGPbqAcqGHiiIZcqWFjbqsdaWgaaadbaGaem4AaSgabeaaaSqab0GaeyyeIuoaaaa@44A8@. Then, the likelihood of an arbitrary measurement *x*_*ij *_relative to this mean expression level is

p(xij)=12π(σj2+ε2)exp⁡[−12(xij−x¯jk)2+ε2σj2+ε2],     (1)
 MathType@MTEF@5@5@+=feaafiart1ev1aaatCvAUfKttLearuWrP9MDH5MBPbIqV92AaeXatLxBI9gBaebbnrfifHhDYfgasaacH8akY=wiFfYdH8Gipec8Eeeu0xXdbba9frFj0=OqFfea0dXdd9vqai=hGuQ8kuc9pgc9s8qqaq=dirpe0xb9q8qiLsFr0=vr0=vr0dc8meaabaqaciaacaGaaeqabaqabeGadaaakeaacqWGWbaCcqGGOaakcqWG4baEdaWgaaWcbaGaemyAaKMaemOAaOgabeaakiabcMcaPiabg2da9maalaaabaGaeGymaedabaWaaOaaaeaacqaIYaGmiiGacqWFapaCdaqadaqaaiab=n8aZnaaDaaaleaacqWGQbGAaeaacqaIYaGmaaGccqGHRaWkcqWF1oqzdaahaaWcbeqaaiabikdaYaaaaOGaayjkaiaawMcaaaWcbeaaaaGccyGGLbqzcqGG4baEcqGGWbaCdaWadaqaaiabgkHiTmaalaaabaGaeGymaedabaGaeGOmaidaamaalaaabaGaeiikaGIaemiEaG3aaSbaaSqaaiabdMgaPjabdQgaQbqabaGccqGHsislcuWG4baEgaqeamaaBaaaleaacqWGQbGAcqWGRbWAaeqaaOGaeiykaKYaaWbaaSqabeaacqaIYaGmaaGccqGHRaWkcqWF1oqzdaahaaWcbeqaaiabikdaYaaaaOqaaiab=n8aZnaaDaaaleaacqWGQbGAaeaacqaIYaGmaaGccqGHRaWkcqWF1oqzdaahaaWcbeqaaiabikdaYaaaaaaakiaawUfacaGLDbaacqGGSaalcaWLjaGaaCzcamaabmaabaGaeGymaedacaGLOaGaayzkaaaaaa@688B@

which includes the term *ε *for an unknown systematic error in condition *j*, here assumed to be the same for all *j*. Note that the use of *σ*_*j *_over all genes **I **rather than a *σ*_*jk *_computed over **I**_*k *_results in a lower likelihood *p*(*x*_*ij*_) for those conditions *j *that have a small overall variance, and are therefore more likely to be correlated by random chance. Also, such low-variance conditions could be the result of poor labeling, or other systematic problems.

The likelihood of the measurements of an arbitrary gene *i *among the conditions in bicluster *k *are p(xi)=∏j∈Jkp(xij)
MathType@MTEF@5@5@+=feaafiart1ev1aaatCvAUfKttLearuWrP9MDH5MBPbIqV92AaeXatLxBI9gBaebbnrfifHhDYfgasaacH8akY=wiFfYdH8Gipec8Eeeu0xXdbba9frFj0=OqFfea0dXdd9vqai=hGuQ8kuc9pgc9s8qqaq=dirpe0xb9q8qiLsFr0=vr0=vr0dc8meaabaqaciaacaGaaeqabaqabeGadaaakeaacqWGWbaCcqGGOaakcqWG4baEdaWgaaWcbaGaemyAaKgabeaakiabcMcaPiabg2da9maarababaGaemiCaaNaeiikaGIaemiEaG3aaSbaaSqaaiabdMgaPjabdQgaQbqabaGccqGGPaqkaSqaaiabdQgaQjabgIGioJqabiab=PeaknaaBaaameaacqWGRbWAaeqaaaWcbeqdcqGHpis1aaaa@42C6@, and similarly the likelihood of a condition *j*'s measurements are p(xj)=∏i∈Ikp(xij)
MathType@MTEF@5@5@+=feaafiart1ev1aaatCvAUfKttLearuWrP9MDH5MBPbIqV92AaeXatLxBI9gBaebbnrfifHhDYfgasaacH8akY=wiFfYdH8Gipec8Eeeu0xXdbba9frFj0=OqFfea0dXdd9vqai=hGuQ8kuc9pgc9s8qqaq=dirpe0xb9q8qiLsFr0=vr0=vr0dc8meaabaqaciaacaGaaeqabaqabeGadaaakeaacqWGWbaCcqGGOaakcqWG4baEdaWgaaWcbaGaemOAaOgabeaakiabcMcaPiabg2da9maarababaGaemiCaaNaeiikaGIaemiEaG3aaSbaaSqaaiabdMgaPjabdQgaQbqabaGccqGGPaqkaSqaaiabdMgaPjabgIGioJqabiab=LeajnaaBaaameaacqWGRbWAaeqaaaWcbeqdcqGHpis1aaaa@42C4@. We integrate the two tails of the Normal distribution in Eq. 1 to derive *co-expression p*-values for each gene *i, r*_*ik*_, and for each condition *j, r*_*jk*_, relative to bicluster *k*.

#### Sequence component (motif co-occurrence)

Each gene *i *has an upstream cis-regulatory sequence *S*_*i *_(a string of DNA nucleotides of length *l*_S_), and bicluster *k *defines a set of sequences **S**_*k *_for all *S*_*i'*_; *i*' ∈ **I**_*k*_. The decision whether an arbitrary gene's upstream sequence, *S*_*i*_, shares common motif(s) with sequences **S**_*k*_, is determined via a two-step process: (1) identify one or more motif(s) **M**_*k *_that is (are) significantly overrepresented in many (if not all) bicluster sequences **S**_*k*_, and then (2) scan *Si *to see if it also contains **M**_*k*_.

In this work, we are not advancing the basic methodology for motif detection (step 1), as relatively mature methods exist for finding motifs given a fixed set of sequences [91]. Instead, we are describing an overall strategy that incorporates previously existing motif finding algorithm(s) into a clustering procedure. As such, the procedure is motif-detection-algorithm agnostic, and the search may be performed using one of many existing methods [91]. Our only requirements are that (a) significantly overrepresented motifs do not have to exist in *all *sequences **S**_*k*_, and (b) it can produce a score (preferentially a *p*-value) that an arbitrary sequence contains the detected motif(s). The MEME algorithm [10], which identifies significant sequence motifs using expectation maximization of one or more probabilistic motif models given a fixed set of sequences and a background residue model, is used to perform step (1), as it meets the first criterion (a). MEME's companion algorithm MAST [10], which computes the *p*-value that an arbitrary sequence matches the set of motifs detected with MEME, is used to perform step (2), as it meets the second criterion (b). During the motif detection step, for any genes in bicluster *k *which are in an operon, we make sure to use only *one copy *of the upstream sequence for that operon (*i.e*. upstream of the first gene in that operon), as described above ("Upstream sequences"). Additional details on the specific parameters passed to these procedures are provided in [Additional File 1, Table Three].

Thus, using these two algorithms, we can detect a set of motifs **M**_*k *_in sequences **S**_*k*_, and compute a *p*-value that a sequence *S*_*i *_contains those motifs. Note that this *p*-value is computed for *each *upstream sequence in the genome, including those for the genes *within *cluster *k*, to derive the *motif p*-values, *s*_*ik*_, for each gene *i *relative to bicluster *k*, at each iteration of the MCMC procedure.

#### Association network component

To build up a highly-connected subnetwork among genes that are in a bicluster (given a full set of associations), we aim to add genes preferentially that have a greater number of connections to those currently in the bicluster than one would expect (at random) based upon the overall connectivity in the network. Thus, we compute *p*-values for observing the associations between a gene or experimental condition and the genes or conditions currently in bicluster *k*, given an association network *N *∈ **N**. In the following discussion, genes are the primary consideration, but networks of associations between experimental conditions are conceivable (*e.g*., we might wish to preferentially group conditions that are part of the same time series). The *network association p*-value, qikN
 MathType@MTEF@5@5@+=feaafiart1ev1aaatCvAUfKttLearuWrP9MDH5MBPbIqV92AaeXatLxBI9gBaebbnrfifHhDYfgasaacH8akY=wiFfYdH8Gipec8Eeeu0xXdbba9frFj0=OqFfea0dXdd9vqai=hGuQ8kuc9pgc9s8qqaq=dirpe0xb9q8qiLsFr0=vr0=vr0dc8meaabaqaciaacaGaaeqabaqabeGadaaakeaacqWGXbqCdaqhaaWcbaGaemyAaKMaem4AaSgabaGaemOta4eaaaaa@3223@, is computed based upon the number of edges in network *N *connecting gene *i *to genes **I**_*k *_in bicluster *k*, relative to the total number of edges connected between *i *and the genes I′k
 MathType@MTEF@5@5@+=feaafiart1ev1aaatCvAUfKttLearuWrP9MDH5MBPbIqV92AaeXatLxBI9gBaebbnrfifHhDYfgasaacH8akY=wiFfYdH8Gipec8Eeeu0xXdbba9frFj0=OqFfea0dXdd9vqai=hGuQ8kuc9pgc9s8qqaq=dirpe0xb9q8qiLsFr0=vr0=vr0dc8meaabaqaciaacaGaaeqabaqabeGadaaakeaaieqacuWFjbqsgaqbamaaBaaaleaacqWGRbWAaeqaaaaa@2F64@ (that are *not *in cluster *k*), as well as the connections within and between the gene sets **I**_*k *_and I′k
 MathType@MTEF@5@5@+=feaafiart1ev1aaatCvAUfKttLearuWrP9MDH5MBPbIqV92AaeXatLxBI9gBaebbnrfifHhDYfgasaacH8akY=wiFfYdH8Gipec8Eeeu0xXdbba9frFj0=OqFfea0dXdd9vqai=hGuQ8kuc9pgc9s8qqaq=dirpe0xb9q8qiLsFr0=vr0=vr0dc8meaabaqaciaacaGaaeqabaqabeGadaaakeaaieqacuWFjbqsgaqbamaaBaaaleaacqWGRbWAaeqaaaaa@2F64@. The hypergeometric distribution is used to compute the probability of observing such an arrangement of connections by chance:

p(ni→Ik|ni→I′k,nIk→Ik,nIk→I′k)=(ni→Ik+nIk→Ikni→Ik)(ni→I′k+nIk→I′kni→I′k)(ni→Ik+nIk→Ik+ni→I′k+nIk→I′kni→Ik+ni→I′k),     (2)
 MathType@MTEF@5@5@+=feaafiart1ev1aaatCvAUfKttLearuWrP9MDH5MBPbIqV92AaeXatLxBI9gBaebbnrfifHhDYfgasaacH8akY=wiFfYdH8Gipec8Eeeu0xXdbba9frFj0=OqFfea0dXdd9vqai=hGuQ8kuc9pgc9s8qqaq=dirpe0xb9q8qiLsFr0=vr0=vr0dc8meaabaqaciaacaGaaeqabaqabeGadaaakeaacqWGWbaCcqGGOaakcqWGUbGBdaWgaaWcbaGaemyAaKMaeyOKH4kcbeGae8xsaK0aaSbaaWqaaiabdUgaRbqabaaaleqaaOGaeiiFaWNaemOBa42aaSbaaSqaaiabdMgaPjabgkziUkqb=LeajzaafaWaaSbaaWqaaiabdUgaRbqabaaaleqaaOGaeiilaWIaemOBa42aaSbaaSqaaiab=LeajnaaBaaameaacqWGRbWAaeqaaSGaeyOKH4Qae8xsaK0aaSbaaWqaaiabdUgaRbqabaaaleqaaOGaeiilaWIaemOBa42aaSbaaSqaaiab=LeajnaaBaaameaacqWGRbWAaeqaaSGaeyOKH4Qaf8xsaKKbauaadaWgaaadbaGaem4AaSgabeaaaSqabaGccqGGPaqkcqGH9aqpdaWcaaqaamaabmaabaqbaeqabiqaaaqaaiabd6gaUnaaBaaaleaacqWGPbqAcqGHsgIRcqWFjbqsdaWgaaadbaGaem4AaSgabeaaaSqabaGccqGHRaWkcqWGUbGBdaWgaaWcbaGae8xsaK0aaSbaaWqaaiabdUgaRbqabaWccqGHsgIRcqWFjbqsdaWgaaadbaGaem4AaSgabeaaaSqabaaakeaacqWGUbGBdaWgaaWcbaGaemyAaKMaeyOKH4Qae8xsaK0aaSbaaWqaaiabdUgaRbqabaaaleqaaaaaaOGaayjkaiaawMcaamaabmaabaqbaeqabiqaaaqaaiabd6gaUnaaBaaaleaacqWGPbqAcqGHsgIRcuWFjbqsgaqbamaaBaaameaacqWGRbWAaeqaaaWcbeaakiabgUcaRiabd6gaUnaaBaaaleaacqWFjbqsdaWgaaadbaGaem4AaSgabeaaliabgkziUkqb=LeajzaafaWaaSbaaWqaaiabdUgaRbqabaaaleqaaaGcbaGaemOBa42aaSbaaSqaaiabdMgaPjabgkziUkqb=LeajzaafaWaaSbaaWqaaiabdUgaRbqabaaaleqaaaaaaOGaayjkaiaawMcaaaqaamaabmaabaqbaeqabiqaaaqaaiabd6gaUnaaBaaaleaacqWGPbqAcqGHsgIRcqWFjbqsdaWgaaadbaGaem4AaSgabeaaaSqabaGccqGHRaWkcqWGUbGBdaWgaaWcbaGae8xsaK0aaSbaaWqaaiabdUgaRbqabaWccqGHsgIRcqWFjbqsdaWgaaadbaGaem4AaSgabeaaaSqabaGccqGHRaWkcqWGUbGBdaWgaaWcbaGaemyAaKMaeyOKH4Qaf8xsaKKbauaadaWgaaadbaGaem4AaSgabeaaaSqabaGccqGHRaWkcqWGUbGBdaWgaaWcbaGae8xsaK0aaSbaaWqaaiabdUgaRbqabaWccqGHsgIRcuWFjbqsgaqbamaaBaaameaacqWGRbWAaeqaaaWcbeaaaOqaaiabd6gaUnaaBaaaleaacqWGPbqAcqGHsgIRcqWFjbqsdaWgaaadbaGaem4AaSgabeaaaSqabaGccqGHRaWkcqWGUbGBdaWgaaWcbaGaemyAaKMaeyOKH4Qaf8xsaKKbauaadaWgaaadbaGaem4AaSgabeaaaSqabaaaaaGccaGLOaGaayzkaaaaaiabcYcaSiaaxMaacaWLjaWaaeWaaeaacqaIYaGmaiaawIcacaGLPaaaaaa@C436@

where **A **→ **B **represents the set of associations between the elements in gene set **A **with those in set **B**, and *n*_**A**→**B **_is the number of these associations. Expression (2) is analogous to the hypergeometric measure of mutual clustering coefficient described by [37]. However, it does not account for the global structure of the network; it is only concerned with the local associations, *i.e*. those directly connected to gene *i *and the bicluster's genes, **I**_*k*_. This choice of connectivity measure allows a single value to be directly computed for each gene, relative to each cluster, and gives greater preference to an individual gene *i *being added to cluster *k *if a large fraction of *i's *associations are with the other genes in the cluster (and vice versa), independent of the global distribution of associations in the network. Individual *p*-values, qikN
 MathType@MTEF@5@5@+=feaafiart1ev1aaatCvAUfKttLearuWrP9MDH5MBPbIqV92AaeXatLxBI9gBaebbnrfifHhDYfgasaacH8akY=wiFfYdH8Gipec8Eeeu0xXdbba9frFj0=OqFfea0dXdd9vqai=hGuQ8kuc9pgc9s8qqaq=dirpe0xb9q8qiLsFr0=vr0=vr0dc8meaabaqaciaacaGaaeqabaqabeGadaaakeaacqWGXbqCdaqhaaWcbaGaemyAaKMaem4AaSgabaGaemOta4eaaaaa@3223@, for each gene *i *and each network *N *are computed for bicluster *k *by integrating the lower tail of the distribution in Eq. 2.

#### The joint cluster membership probability

The ultimate goal is to decide gene or condition bicluster membership jointly on the basis of the three individual sets of *p*-values *r*_*ik*_, *s*_*ik*_, and *q*_*ik *_computed above (for the remainder of this discussion, we now use *i *to denote a gene *or *experimental condition). A common procedure for combining "scores" such as these into a single joint likelihood is to perform a multi-parametric logistic regression [41] that treats each *p*-value measure as a random variable and estimates the joint membership likelihood *p*(*y*_*ik *_= 1) using the logistic function,

πik≡p(yik=1|Xk,Si,Mk,N)∝exp⁡[β0+r0log⁡(rik)+s0log⁡(sik)+∑N∈Nq0Nlog⁡(qikN)].     (3)
 MathType@MTEF@5@5@+=feaafiart1ev1aaatCvAUfKttLearuWrP9MDH5MBPbIqV92AaeXatLxBI9gBaebbnrfifHhDYfgasaacH8akY=wiFfYdH8Gipec8Eeeu0xXdbba9frFj0=OqFfea0dXdd9vqai=hGuQ8kuc9pgc9s8qqaq=dirpe0xb9q8qiLsFr0=vr0=vr0dc8meaabaqaciaacaGaaeqabaqabeGadaaakeaaiiGacqWFapaCdaWgaaWcbaGaemyAaKMaem4AaSgabeaakiabggMi6kabdchaWjabcIcaOiabdMha5naaBaaaleaacqWGPbqAcqWGRbWAaeqaaOGaeyypa0JaeGymaeJaeiiFaWhcbeGae4hwaG1aaSbaaSqaaiabdUgaRbqabaGccqGGSaalcqWGtbWudaWgaaWcbaGaemyAaKgabeaakiabcYcaSiab+1eannaaBaaaleaacqWGRbWAaeqaaOGaeiilaWIae4Nta4KaeiykaKIaeyyhIuRagiyzauMaeiiEaGNaeiiCaa3aamWaaeaacqWFYoGydaWgaaWcbaGaeGimaadabeaakiabgUcaRiabdkhaYnaaBaaaleaacqaIWaamaeqaaOGagiiBaWMaei4Ba8Maei4zaCMaeiikaGIaemOCai3aaSbaaSqaaiabdMgaPjabdUgaRbqabaGccqGGPaqkcqGHRaWkcqWGZbWCdaWgaaWcbaGaeGimaadabeaakiGbcYgaSjabc+gaVjabcEgaNjabcIcaOiabdohaZnaaBaaaleaacqWGPbqAcqWGRbWAaeqaaOGaeiykaKIaey4kaSYaaabuaeaacqWGXbqCdaqhaaWcbaGaeGimaadabaGaemOta4eaaaqaaiabd6eaojabgIGiolab+5eaobqab0GaeyyeIuoakiGbcYgaSjabc+gaVjabcEgaNjabcIcaOiabdghaXnaaDaaaleaacqWGPbqAcqWGRbWAaeaacqWGobGtaaGccqGGPaqkaiaawUfacaGLDbaacqGGUaGlcaWLjaGaaCzcamaabmaabaGaeG4mamdacaGLOaGaayzkaaaaaa@8A43@

This model approximates a (probabilistic) discriminating hyperplane in the space defined by *r*_*ik*_, *s*_*ik*_, and *q*_*ik*_, parameterized by the four independent variables *β*_0 _(the intercept), and *r*_0_, *s*_0_, and *q*_0 _(the slope) that maximally discriminates the genes or conditions within the bicluster (**I**_*k*_) from those outside (I′k
 MathType@MTEF@5@5@+=feaafiart1ev1aaatCvAUfKttLearuWrP9MDH5MBPbIqV92AaeXatLxBI9gBaebbnrfifHhDYfgasaacH8akY=wiFfYdH8Gipec8Eeeu0xXdbba9frFj0=OqFfea0dXdd9vqai=hGuQ8kuc9pgc9s8qqaq=dirpe0xb9q8qiLsFr0=vr0=vr0dc8meaabaqaciaacaGaaeqabaqabeGadaaakeaaieqacuWFjbqsgaqbamaaBaaaleaacqWGRbWAaeqaaaaa@2F64@). Conceptually, the model implies that a gene or condition that poorly matches the bicluster based on one data type can still be added to the bicluster if it matches well to the other data types, analogous to, for example, the explicit "softening" of cluster boundaries performed by [15]. Note that when element *i *is an experimental condition, *s*_0 _(the motif parameter) is zero.

In practice, during early iterations when the bicluster is not well-discriminated from the background, such an unconstrained regression leads to unstable situations such as unwarranted over-weighting or inversion of one or more variables (*r*_0_, *s*_0_, or *q*_0_). Additionally, depending upon the quality of the data set(s) being used and the predisposition (or prior knowledge) of the researcher, different runs of the algorithm stressing different data types may be desired. Finally, there is good reason to expect that certain data types (*e.g*. sequence motifs) will be less informative early in the procedure when the biclusters are poorly-defined, and only later will it make sense to incorporate them into the bicluster model.

Therefore, we perform a *constrained *logistic regression by transforming the regression space defined by *r*_*ik*_, *s*_*ik*_, and *q*_*ik *_into one dimension, projecting the log-*p*-values onto a single vector, *g*_*ik*_,

gik=r0log⁡(r˜ik)+s0log⁡(s˜ik)+∑N∈Nq0Nlog⁡(q˜ikN),     (4)
 MathType@MTEF@5@5@+=feaafiart1ev1aaatCvAUfKttLearuWrP9MDH5MBPbIqV92AaeXatLxBI9gBaebbnrfifHhDYfgasaacH8akY=wiFfYdH8Gipec8Eeeu0xXdbba9frFj0=OqFfea0dXdd9vqai=hGuQ8kuc9pgc9s8qqaq=dirpe0xb9q8qiLsFr0=vr0=vr0dc8meaabaqaciaacaGaaeqabaqabeGadaaakeaacqWGNbWzdaWgaaWcbaGaemyAaKMaem4AaSgabeaakiabg2da9iabdkhaYnaaBaaaleaacqaIWaamaeqaaOGagiiBaWMaei4Ba8Maei4zaCMaeiikaGIafmOCaiNbaGaadaWgaaWcbaGaemyAaKMaem4AaSgabeaakiabcMcaPiabgUcaRiabdohaZnaaBaaaleaacqaIWaamaeqaaOGagiiBaWMaei4Ba8Maei4zaCMaeiikaGIafm4CamNbaGaadaWgaaWcbaGaemyAaKMaem4AaSgabeaakiabcMcaPiabgUcaRmaaqafabaGaemyCae3aa0baaSqaaiabicdaWaqaaiabd6eaobaakiGbcYgaSjabc+gaVjabcEgaNjabcIcaOiqbdghaXzaaiaWaa0baaSqaaiabdMgaPjabdUgaRbqaaiabd6eaobaakiabcMcaPiabcYcaSiaaxMaacaWLjaWaaeWaaeaacqaI0aanaiaawIcacaGLPaaaaSqaaiabd6eaojabgIGioJqabiab=5eaobqab0GaeyyeIuoaaaa@670A@

where *r*_0_, *s*_0_, and *q*_0 _are specified for each iteration according to an "annealing schedule," described below. Here, each of the dimensions have been standardized to place the log-*p*-values for each data set on the same scale, with log(χ˜
 MathType@MTEF@5@5@+=feaafiart1ev1aaatCvAUfKttLearuWrP9MDH5MBPbIqV92AaeXatLxBI9gBaebbnrfifHhDYfgasaacH8akY=wiFfYdH8Gipec8Eeeu0xXdbba9frFj0=OqFfea0dXdd9vqai=hGuQ8kuc9pgc9s8qqaq=dirpe0xb9q8qiLsFr0=vr0=vr0dc8meaabaqaciaacaGaaeqabaqabeGadaaakeaacuaHhpWygaacaaaa@2E72@_*ik*_) = [log(*χ*_*ik *_) - *μ*_*k*_]/*σ*_*k*_, where *μ*_*k *_and *σ*_*k *_are the mean and standard deviation of the log-*p*-values *log*(*χ*_*ik*_) (*χ*_*ik *_denotes either *r*_*ik*_, *s*_*ik*_, or *q*_*ik*_), only over the genes or conditions in the bicluster (*i ∈ *I_*k*_). This is necessary because the *p*-values for each component of our model were derived for different types of data, each with widely differing sizes. For example, the *p*-values are likely to be smaller (on average) for the component with the most data (here, the expression data), or for motifs with larger lengths. The projection described in Eq. 4 constrains the regression by fixing the slope of the discriminating hyperplane (via parameters *r*_0_, *s*_0_, and *q*_0_), with only the intercept *β*_0 _permitted to vary from cluster to cluster. These parameters may also be interpreted as mixing parameters that control the fractional contribution of each model component to the cluster likelihood, *g*_*ik*_. They may be defined by the user, and/or may be modified throughout the course of cluster optimization. For example, early in the procedure when the bicluster is a poorly-defined seed, co-expression and certain association networks (*e.g*. operon associations, for bacteria and archaea) are extremely informative, whereas a common cis-regulatory motif is less likely exist among the genes in the bicluster. Only later (when the bicluster has been optimized on the basis of expression data and operon associations) does it make sense to incorporate sequence motifs into the bicluster model. Therefore we employ a strategy for slowly varying the relative contribution of each of the regression parameters, as the cluster is optimized, as part of an annealing schedule (described further, below). The constrained binomial regression is now given by

*π*_*ik *_≡ *p*(*y*_*ik *_= 1|**X**_*k*_, *S*_*i*_, **M**_*k*_, **N**) ∝ exp(*β*_0 _+ *β*_1_g_*ik*_),     (5)

where parameters ***β ***= [*β*_0_, *β*_1_] fully determine the conditional probability of membership *p*(*y*_*ik *_= 1) of a gene or condition *i *in bicluster *k*.

One additional complication arises near the end of a bicluster optimization, that a bicluster may be perfectly discriminated from the background (resulting in an infinite negative log-likelihood and undefined regression). This may be addressed in two ways: the first is to constrain or fix the slope *β*_1 _of the regression, allowing only the intercept *β*_0 _to vary. We chose a second option, to perform a penalized maximum likelihood estimation described by [42] and originally proposed by [35]. This penalized estimate of *β *provides bias reduction in the case of small sample sizes (small biclusters), and solves the separation problem in the context of perfect discrimination and infinite likelihood. *β *can be determined with this penalized likelihood measure using an efficient iterative process [35].

We now have a set of probabilities, *π*_*ik*_, that each gene or condition *i *is associated with bicluster *k *given the bicluster's current state. We would now like to perform moves (*i.e*. add or remove genes and conditions) that are most likely to improve the likelihood of the bicluster based upon the model. We do this by sampling moves from *π*_*ik*_. These probabilities may be further adjusted via additional (prior) constraints on the model, as described below.

### The cMonkey iterative procedure

#### Seeding the clusters

The Markov chain process by which a bicluster is optimized requires "seeding" of the bicluster to start the procedure. We experimented with many data-driven methods for generating seed biclusters, including (a) single-gene seeds, (b) random or semi-correlated seeds using a pre-specified distribution of cluster sizes, and (c) seeding on the basis of co-expressed edges in association networks (for example, operon associations). In principle, any seeding method may be used, including the clusters produced by other clustering or biclustering methods. Many *different *seeding methods are used in order to broaden the parameter space which is searched, and depending upon the annealing schedule used, the algorithm can be made more- or less-sensitive to the selected starting points. As biclusters are optimized sequentially, in order to maximize our coverage of the overall (gene) search space, they are seeded only with genes that have not previously been placed into any other biclusters. It should be noted, however, that during subsequent iterations, genes that are already in other biclusters *can *still be added to new biclusters, with additional constraints that are described later.

Each bicluster is seeded using a random choice from one of a variety of methods, each of which utilizes one or more different types of input data. For each newly-seeded bicluster, **I**' be the set of genes that are currently not in any other biclusters, *i *is a randomly-chosen gene from **I**' and **J**_*i *_is the set of conditions in which *i *has the highest amount of variance. The seeding methods available are:

1. *A single random gene*: The cluster is seeded with *i *and **J**_*i*_. For the first few iterations of this bicluster's optimization, only gene additions are allowed (forcing the bicluster to grow in size, early on).

3. *n co-expressed genes from another clustering method*: Clusters are generated using an other clustering or biclustering method, and these are used as seeds for further optimization.

2. *n semi-co-expressed genes*: Up to *n *- 1 additional genes from **I**' are randomly chosen from those with a high Pearson correlation (*P*_*c *_> 0.8) with *i *in conditions **J**_*i*_. n is chosen randomly from a set of pre-defined cluster seeding sizes, currently 2, 5, 10, 20, *μ*, where *μ*, is described Methods.

4. *n highly-connected genes*: Up to *n *- 1 random genes from **I**' are added from those with *P*_*c *_> 0 with *i*, and are first neighbors with *i *in a given association network, *n *is chosen as in (2); the association network is chosen randomly from the following (if available for that organism): operons, metabolic assoc., protein-DNA interactions, protein-protein interactions.

5. *n genes with a common motif*: Up to *n *- 1 genes from **I**' are randomly chosen from those with *P*_*c *_> 0 with *i*, and also have a common *d*-mer with *i *in their upstream sequences, allowing for up to *l *residue differences, *n *is chosen as in (2); defaults of *d *= 9 and *l *= 1 were used.

#### Annealing the clusters

A newly-seeded bicluster *k *is iteratively improved with respect to the joint likelihood derived above. At each iteration, significant motifs are detected (using MEME), and the joint membership probabilities *π*_*ik *_for each gene or condition *i *are computed. We then perform moves using Simulated Annealing (SA) [51], to preferentially add genes or conditions *i *to bicluster *k *if they have a high probability of membership (*y*_*ik *_= 0 and *π*_*ik *_≈ 1), and to drop genes or conditions from that bicluster if they have a low probability of membership (*y*_*ik *_= 1 and *π*_*ik *_≈ 0). Moves which may decrease the likelihood of the cluster model are permitted, with a frequency that decreases during the course of the procedure, as parameterized by an annealing temperature *T*:

p(add|πik)=e−πik/T;p(drop|πik)=e−(1−πik)/T.     (6)
 MathType@MTEF@5@5@+=feaafiart1ev1aaatCvAUfKttLearuWrP9MDH5MBPbIqV92AaeXatLxBI9gBaebbnrfifHhDYfgasaacH8akY=wiFfYdH8Gipec8Eeeu0xXdbba9frFj0=OqFfea0dXdd9vqai=hGuQ8kuc9pgc9s8qqaq=dirpe0xb9q8qiLsFr0=vr0=vr0dc8meaabaqaciaacaGaaeqabaqabeGadaaakeaafaqabeqacaaabaGaemiCaaNaeiikaGccbaGae8xyaeMae8hzaqMae8hzaqMaeiiFaWhcciGae4hWda3aaSbaaSqaaiabdMgaPjabdUgaRbqabaGccqGGPaqkcqGH9aqpcqWGLbqzdaahaaWcbeqaaiabgkHiTiab+b8aWnaaBaaameaacqWGPbqAcqWGRbWAaeqaaSGaei4la8IaemivaqfaaOGaei4oaSdabaGaemiCaaNaeiikaGIae8hzaqMae8NCaiNae83Ba8Mae8hCaaNaeiiFaWNae4hWda3aaSbaaSqaaiabdMgaPjabdUgaRbqabaGccqGGPaqkcqGH9aqpcqWGLbqzdaahaaWcbeqaaiabgkHiTiabcIcaOiabigdaXiabgkHiTiab+b8aWnaaBaaameaacqWGPbqAcqWGRbWAaeqaaSGaeiykaKIaei4la8Iaemivaqfaaaaakiabc6caUiaaxMaacaWLjaWaaeWaaeaacqaI2aGnaiaawIcacaGLPaaaaaa@6663@

All moves are performed by sampling them from the probability in Eq. 6. This Simulated Annealing procedure is dampened by restricting the total number of gene/condition moves at each iteration to *n*_max _= 5, in order to reduce the chance that a bicluster will change drastically before its model is reevaluated. We find that Simulated Annealing, while not the most efficient search strategy available, improves upon greedy search strategies such as Expectation Maximization, by being able to escape local minima and therefore being able to more completely assign genes and conditions to clusters as appropriate [24]. Other stochastic or greedy search strategies may be applicable to solving this model, for example if speed is deemed to be a more important consideration than completeness of the solution.

#### Additional model constraints: bicluster size and overlap

The search space for this problem is often dominated by very strong attractors and if we do not restrict the gene/condition move set, biclusters are likely to repeatedly descend into the same set of deep local minima (thereby increasing the bicluster overlap, or redundancy). This is an issue seen in many biclustering algorithms, and a commonly-practiced *ad hoc *remedy is to post-filter the bicluster set to remove redundant ones. We choose a more straightforward, easily-parameterized solution: to constrain the total number of biclusters *z*_*i *_into which each gene *i *may fall (and in effect to reduce the amount of "gene overlap" of the final bicluster set), *z*_*i *_is modeled as a Poisson process with cumulative distribution *F*_*v*_(z_*i*_) (where *v *is the expected number of biclusters per gene). Then the probability of adding or dropping *i *to/from bicluster *k*, *p*(add|*π*_*ik*_) and *p*(drop|*π*_*ik*_) (Eq. 6), is multiplied with this prior probability of observing the gene in that many biclusters (relative to the expected number):

p′(add|πik)=Fv(zi)/Fv(v)e−πik/T;p′(drop|πik)=[1−Fv(zi)]/[1−Fv(v)]e−(1−πik)/T.     (7)
 MathType@MTEF@5@5@+=feaafiart1ev1aaatCvAUfKttLearuWrP9MDH5MBPbIqV92AaeXatLxBI9gBaebbnrfifHhDYfgasaacH8akY=wiFfYdH8Gipec8Eeeu0xXdbba9frFj0=OqFfea0dXdd9vqai=hGuQ8kuc9pgc9s8qqaq=dirpe0xb9q8qiLsFr0=vr0=vr0dc8meaabaqaciaacaGaaeqabaqabeGadaaakeaafaqadeGadaaabaGafmiCaaNbauaacqGGOaakieaacqWFHbqycqWFKbazcqWFKbazcqGG8baFiiGacqGFapaCdaWgaaWcbaGaemyAaKMaem4AaSgabeaakiabcMcaPaqaaiabg2da9aqaaiabdAeagnaaBaaaleaacqWG2bGDaeqaaOGaeiikaGIaemOEaO3aaSbaaSqaaiabdMgaPbqabaGccqGGPaqkcqGGVaWlcqWGgbGrdaWgaaWcbaGaemODayhabeaakiabcIcaOiabdAha2jabcMcaPiabdwgaLnaaCaaaleqabaGaeyOeI0Iae4hWda3aaSbaaWqaaiabdMgaPjabdUgaRbqabaWccqGGVaWlcqWGubavaaGccqGG7aWoaeaacuWGWbaCgaqbaiabcIcaOiab=rgaKjab=jhaYjab=9gaVjab=bhaWjabcYha8jab+b8aWnaaBaaaleaacqWGPbqAcqWGRbWAaeqaaOGaeiykaKcabaGaeyypa0dabaGaei4waSLaeGymaeJaeyOeI0IaemOray0aaSbaaSqaaiabdAha2bqabaGccqGGOaakcqWG6bGEdaWgaaWcbaGaemyAaKgabeaakiabcMcaPiabc2faDjabc+caViabcUfaBjabigdaXiabgkHiTiabdAeagnaaBaaaleaacqWG2bGDaeqaaOGaeiikaGIaemODayNaeiykaKIaeiyxa0Laemyzau2aaWbaaSqabeaacqGHsislcqGGOaakcqaIXaqmcqGHsislcqGFapaCdaWgaaadbaGaemyAaKMaem4AaSgabeaaliabcMcaPiabc+caViabdsfaubaakiabc6caUaaacaWLjaGaaCzcauaabmqabeaaaeaadaqadaqaaiabiEda3aGaayjkaiaawMcaaaaaaaa@8BE6@

Thus the solution is constrained to what seems to be a more biologically intuitive model: include each gene in an average of *v *= 2 (the default) clusters. This constraint results in an increased tendency to drop a gene from a bicluster if it is already in more than two biclusters, and a decreased tendency to drop the gene if it is in less than two biclusters.

Bicluster sizes can also vary widely between biclustering methods; some generate biclusters with only three genes on average [6], to single biclusters with nearly 3/4 of all genes in the data [18]. We constrain bicluster *k*'s final size (number of genes, |**I**_*k*_|), using a cumulative Normal distribution *N*(|**I**_*k*_|, *μ*, *σ*) as a prior constraint on |**I**_*k*_|. This conditional distribution is applied by further adjusting the relative ratios of the distributions (Eq. 6) from which the gene moves are sampled:

∑i∈Ik'p′(drop|πik)∑i∈Ikp′(add|πik)≡N(|Ik|,μ,σ)[1−N(|Ik|,μ,σ)].     (8)
 MathType@MTEF@5@5@+=feaafiart1ev1aaatCvAUfKttLearuWrP9MDH5MBPbIqV92AaeXatLxBI9gBaebbnrfifHhDYfgasaacH8akY=wiFfYdH8Gipec8Eeeu0xXdbba9frFj0=OqFfea0dXdd9vqai=hGuQ8kuc9pgc9s8qqaq=dirpe0xb9q8qiLsFr0=vr0=vr0dc8meaabaqaciaacaGaaeqabaqabeGadaaakeaadaWcaaqaamaaqababaGafmiCaaNbauaacqGGOaakieaacqWFKbazcqWFYbGCcqWFVbWBcqWFWbaCcqGG8baFiiGacqGFapaCdaWgaaWcbaGaemyAaKMaem4AaSgabeaakiabcMcaPaWcbaGaemyAaKMaeyicI4mcbeGae0xsaK0aa0baaWqaaiabdUgaRbqaaiabcEcaNaaaaSqab0GaeyyeIuoaaOqaamaaqababaGafmiCaaNbauaacqGGOaakcqWFHbqycqWFKbazcqWFKbazcqGG8baFcqGFapaCdaWgaaWcbaGaemyAaKMaem4AaSgabeaakiabcMcaPaWcbaGaemyAaKMaeyicI4Sae8xsaK0aaSbaaWqaaiabdUgaRbqabaaaleqaniabggHiLdaaaOGaeyyyIO7aaSaaaeaacqWGobGtcqGGOaakcqGG8baFcqqFjbqsdaWgaaWcbaGaem4AaSgabeaakiabcYha8jabcYcaSiab+X7aTjabcYcaSiab+n8aZjabcMcaPaqaaiabcUfaBjabigdaXiabgkHiTiabd6eaojabcIcaOiabcYha8jab9LeajnaaBaaaleaacqWGRbWAaeqaaOGaeiiFaWNaeiilaWIae4hVd0MaeiilaWIae43WdmNaeiykaKIaeiyxa0faaiabc6caUiaaxMaacaWLjaWaaeWaaeaacqaI4aaoaiaawIcacaGLPaaaaaa@7EE0@

The result is that if |**I**_*k*_| <*μ*, the number of genes sampled to add to the bicluster will tend to be greater than the number sampled to drop, and vice versa if |**I**_*k*_| > *μ*. We parameterize our prior expectation of bicluster sizes using *μ *= *σ *= 30, to match previous estimates of regulon sizes for well-studied organisms (*e.g*. Alkema, Lenhard, and Wasserman, 2004). This amounts to a soft constraint, which still allows for considerable variation in final bicluster sizes. A similar constraint may be applied to the biclusters' experiment sizes, which enables the generation of biclusters with a larger number of experiments (on average) than are typically included in biclusters derived by other methods (*e.g*. [19,86]).

#### The annealing schedule

**Figure 9:**
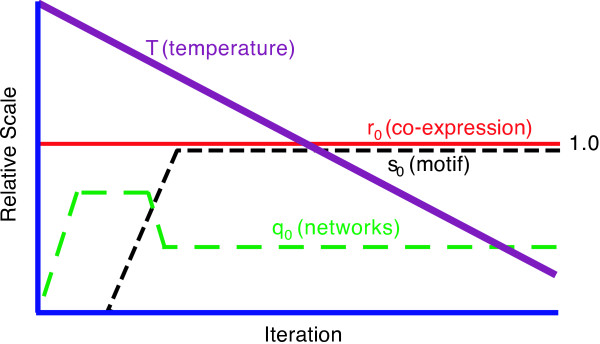
Example annealing schedule applied to the three CMONKEY model component weights (*r*_0_, *p*_0_, and *q*_0_) and annealing temperature *T*, during a bicluster optimization, as a function of iteration.

To enforce convergence we schedule the annealing temperature *T *to slowly decrease during the procedure, as in standard simulated annealing procedures. We find that varying *T *linearly from 0.15 to 0.05 over 100–150 optimization steps is generally effective for all organisms for which biclustering was performed. As was described previously, there are reasons to vary (in addition to *T*) the three model mixture constraint parameters, *r*_0_, *s*_0_, and *q*_0 _with each iteration. We have found that the most effective schedule up-weights the expression (*r*_0_) and certain association networks (*q*_0_; *e.g*. operons and metabolic networks) early in a run to build up co-expressed biclusters, and then slowly increases the influence of the sequence motifs (*s*_0_) as the biclusters become better-defined (Fig. 9). For similar reasons, additional parameters, such as the number of motifs searched for, can also vary (*i.e*. increase or decrease monotonically) with iteration. Details on the default cMonkey parameters used for this work are listed in [Additional File 1, Table Three].

### Implementation

cMonkey is implemented in the *R *statistical programming language [5], a highly-flexible cross-platform language widely used in the statistical community. It has been parallelized, using PVM [84] as implemented in the Simplified Network Of Workstations (*snow*) *R *library, and runs efficiently on a multi-node Linux cluster; it can be run on a single-processor desktop computer as well. On a typical single-2 GHz processor, the algorithm can generate ~100 biclusters in between 12 and 48 hours, depending on the organism, data size, and motif detection parameters chosen. All parameters relevant to the biclustering procedure that have not been described in the main text are listed in [Additional File 1, Table Three].

### Comparison with other biclustering and clustering methods

The different bi/clustering algorithms used for the comparative analysis included: Cheng-Church [25], Order Preserving Submatrix (OPSM [18]), Iterative Signature (ISA [19]), xMOTIF [55], and BIMAX [6] (all of these algorithms were run using the BICAT implementation [17]), SAMBA [86] (as implemented by the authors as part of EXPANDER [77]), and both hierarchical clustering and *k*-means clustering [30] with cluster number (*k*) ranging from 10 to 300 (implemented in *R*). None of these methods utilize data integration, and all were run on the same data sets as cMonkey. All biclustering procedures were run using their default parameters and data normalization/discretization schemes (while the effects of varying the parameters for each of these methods would be a worthwhile study, it is beyond the scope of this work). The analysis was performed on the Gasch [36] subset of the *S. cerevisiae *data containing measurements of 2993 genes over 173 stress-related conditions. *S. cerevisiae *was chosen for these comparisons because of the high-quality data and varied external "references" available for this organism, against which clusters could be compared. In all cases where *p*-values for judging annotation over-representation are listed, they were computed following a procedure similar to [21]; namely, cumulative hypergeometric *p*-values were corrected for multiple hypothesis testing in an *experiment-wise *manner for each cluster, by computing the fraction of uncorrected *p*-values derived for 1000 randomized instances of the cluster (the null model) that were less than or equal to the best *p*-value obtained for that cluster. To assess the effect of various biases caused by inclusion of different parts of the cMonkey model, we performed these same analyses on cMonkey runs with various model parameters up- and down-weighted, as described in the Results section. In all cases where we compared the motif-detection results of specific biclusters (in the Results section), we used MEME and MAST [10] (with the same parameters as for cMonkey) to search for motifs *de novo *in the upstream sequences of the clusters' genes.

Each different biclustering algorithm returned bicluster sets with wide differences in cluster count, cluster size (genes and experiments), amount of overlap/redundancy, expression coherence, and other general characteristics only related to their treatment of the expression data. We therefore computed "bulk" measurements for each bicluster set, such as those listed in [Additional File 1, Table Two]. One of these, *f*, is defined as the total fraction of elements in the expression data matrix **X **which fall in at least one bicluster. A measurement that quantifies the degree to which each complete bicluster set recapitulates the variance in **X **is defined as follows:

RMSD≡∑k∑j∈Jk∑i∈Ik(xij−x¯jk)2nijσ2     (9)
 MathType@MTEF@5@5@+=feaafiart1ev1aaatCvAUfKttLearuWrP9MDH5MBPbIqV92AaeXatLxBI9gBaebbnrfifHhDYfgasaacH8akY=wiFfYdH8Gipec8Eeeu0xXdbba9frFj0=OqFfea0dXdd9vqai=hGuQ8kuc9pgc9s8qqaq=dirpe0xb9q8qiLsFr0=vr0=vr0dc8meaabaqaciaacaGaaeqabaqabeGadaaakeaaieaacqWFsbGucqWFnbqtcqWFtbWucqWFebarcqGHHjIUdaGcaaqaamaaqafabaWaaabuaeaadaaeqbqaamaalaaabaGaeiikaGIaemiEaG3aaSbaaSqaaiabdMgaPjabdQgaQbqabaGccqGHsislcuWG4baEgaqeamaaBaaaleaacqWGQbGAcqWGRbWAaeqaaOGaeiykaKYaaWbaaSqabeaacqaIYaGmaaaakeaacqWGUbGBdaWgaaWcbaGaemyAaKMaemOAaOgabeaaiiGakiab+n8aZnaaCaaaleqabaGaeGOmaidaaaaaaeaacqWGPbqAcqGHiiIZieqacqqFjbqsdaWgaaadbaGaem4AaSgabeaaaSqab0GaeyyeIuoaaSqaaiabdQgaQjabgIGiolab9PeaknaaBaaameaacqWGRbWAaeqaaaWcbeqdcqGHris5aaWcbaGaem4AaSgabeqdcqGHris5aaWcbeaakiaaxMaacaWLjaWaaeWaaeaacqaI5aqoaiaawIcacaGLPaaaaaa@5DCE@

where, as above, x¯jk=∑i∈Ikxij/|Ik|
 MathType@MTEF@5@5@+=feaafiart1ev1aaatCvAUfKttLearuWrP9MDH5MBPbIqV92AaeXatLxBI9gBaebbnrfifHhDYfgasaacH8akY=wiFfYdH8Gipec8Eeeu0xXdbba9frFj0=OqFfea0dXdd9vqai=hGuQ8kuc9pgc9s8qqaq=dirpe0xb9q8qiLsFr0=vr0=vr0dc8meaabaqaciaacaGaaeqabaqabeGadaaakeaacuWG4baEgaqeamaaBaaaleaacqWGQbGAcqWGRbWAaeqaaOGaeyypa0ZaaabeaeaacqWG4baEdaWgaaWcbaGaemyAaKMaemOAaOgabeaaaeaacqWGPbqAcqGHiiIZieqacqWFjbqsdaWgaaadbaGaem4AaSgabeaaaSqab0GaeyyeIuoakiabc+caViabcYha8jab=LeajnaaBaaaleaacqWGRbWAaeqaaOGaeiiFaWhaaa@449D@ is the mean expression profile of bicluster *k*, and *n*_*ij *_= ∑_*k*_*i *∈ **I**_*k *_∧ *j *∈ **J**_*k *_is the number of biclusters containing element *x*_*ij*_. This measure is dependent upon the fractional coverage *f *of the expression data matrix by the bicluster set (better coverage will generally lead to better RMSD) as well as the average bicluster residual (better residual leads to better RMSD), but is nearly independent of bicluster redundancy. It therefore is a good measure of the tradeoff that each bi/clustering method chooses between data coverage and bicluster co-expression. Because the expression data set has been variance-normalized (see Methods), RMSD ranges between 0–1, where a smaller RMSD implies that the mean expression profiles of the biclusters more accurately "generate" the original data matrix **X**.

In an attempt to remove *some *overlap and size bias related to these quality measurements (see Discussion), we also performed tests on a "filtered" set of biclusters, in which we greedily identified the largest 100 clusters with a volume-overlap (genes × conditions) of < 0.5 [6], excluding any with > 200 genes. For methods such as cMonkey, this filtering step removes a large number of non-redundant (but smaller) clusters, while for other methods (*e.g*. OPSM), it removes a large fraction of derived clusters and for others (such as SAMBA) it has little effect. Finally, in a further attempt to disentangle the cluster size bias inherent in these comparisons, we performed the same analyses on a set of evenly divided cluster sets ("big" and "small" halves; results shown in [Additional File 1]).

## Abbreviations

Markov chain Monte Carlo (MCMC), cumulative distribution function (CDF), Position specific scoring matrix (PSSM), Gene Ontology (GO)

## Authors' contributions

**DJR **Developed and implemented the cMonkey algorithm; ran the procedure and analyzed the results; wrote this manuscript.

**NSB **Contributed to the inception of this project; provided important feedback on the results; assisted with the writing of this manuscript.

**RB **Conceived and initiated this project; contributed to the development and implementation of cMonkey; wrote this manuscript.

## Supplementary Material

Additional File 1 Supplementary file containing additional figures and tables, with captions, referenced in the main manuscript. Click here for file

## Figures and Tables

**Table 1:** Means of Pearson correlation coefficients of genes in bR or DMSO putative regulons (rows) with mean profile of genes in bR or DMSO operons (columns) over conditions *within *and *outside *the bicluster.

	bR	DMSO
bR in	0.951	0.866
DMSO in	0.833	0.967
bR out	0.838	0.475
DMSO out	0.442	0.837
