# Supplementary material for: Integrated biclustering of heterogeneous genome-wide datasets for the inference of global regulatory networks
Source: BMC Bioinformatics. 2006 Jun 2;7:280. doi: 10.1186/1471-2105-7-280 (PMC1502140; doi:10.1186/1471-2105-7-280)
Supplement: Additional File 1 — Supplementary file containing additional figures and tables, with captions, referenced in the main manuscript. [file 1471-2105-7-280-S1.pdf]

Supplementary Materials: Additional cMonkey Results and Parameters

Note: Additional files and data, including interactive Cytoscape [78] Java Web Starts of bicluster networks for each organism, and the data that was used to generate those bicluster sets, are available at our cMonkey *web site,* http://halo.systemsbiology.net/cmonkey.

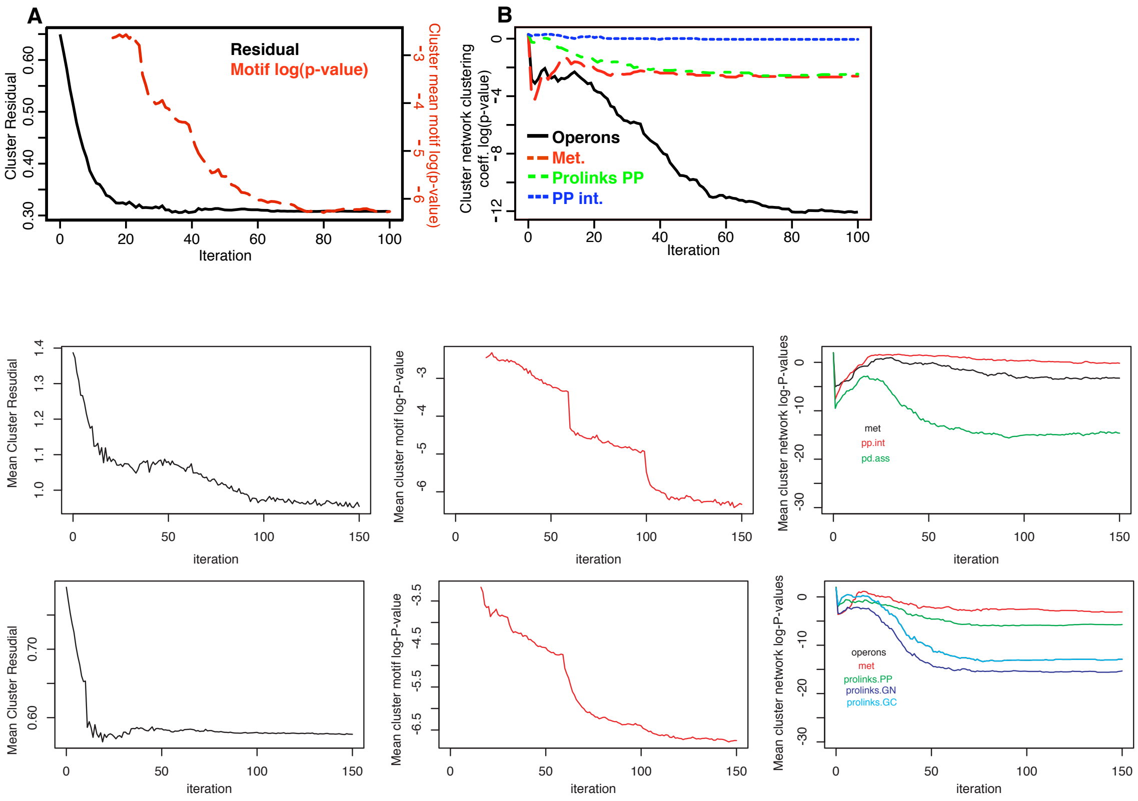

Figure 10. Mean measures of bicluster "quality" with respect to co-expression ("residual," [98]), motif co-occurrence ("Motif log(p-value)"), and mutual clustering coefficient [37] in different association networks, as a function of iteration of bicluster optimization, for *H. pylori* (top), S. cerevisiae (center), and *E. coli (bottom)*. A similar plot is shown for *H. pylori* in Figure 6.

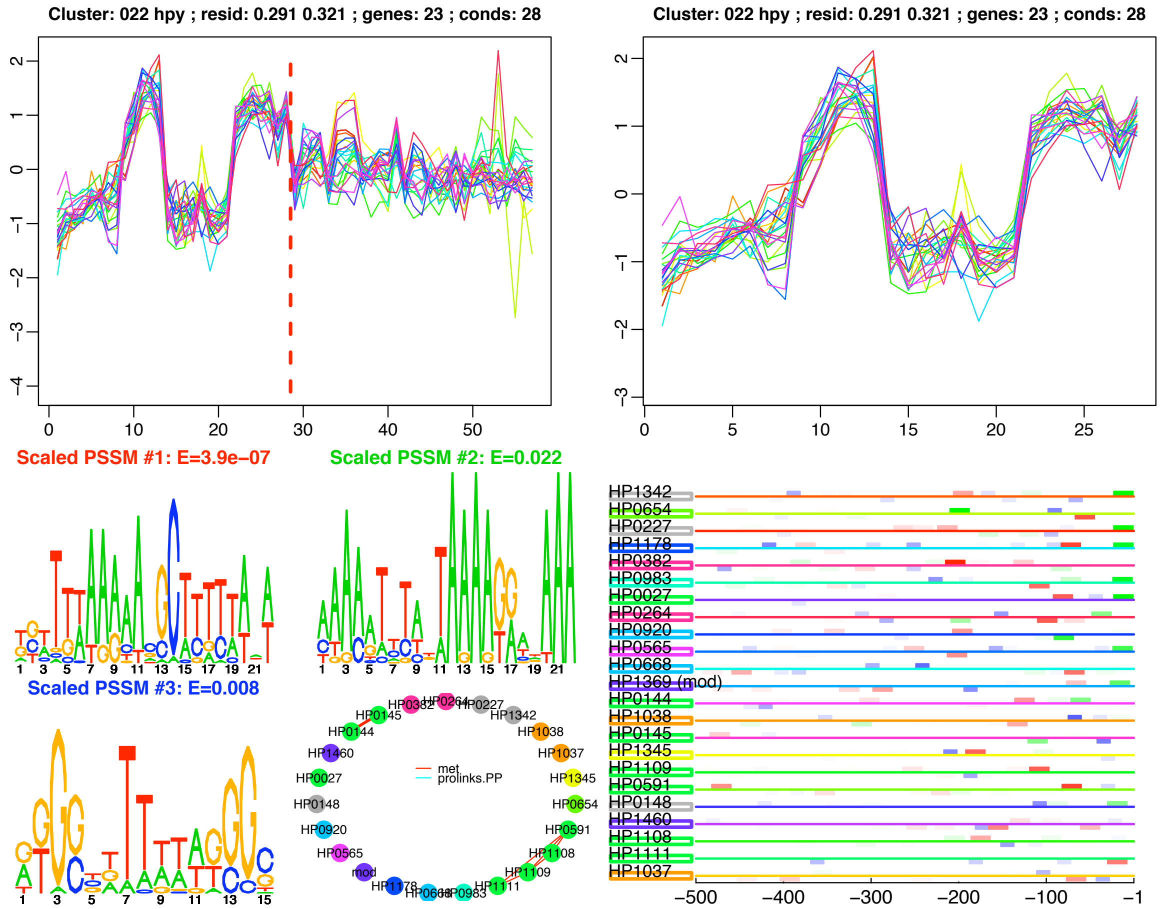

Figure 11. *H. pylori* bicluster containing elements of the electron transport chain and late stages of carbohydrate metabolism as well as several other genes of diverse function including outer membrane protein and membrane proteins of unknown function. The bicluster contains a well-conserved putative semi-palindromic motif.

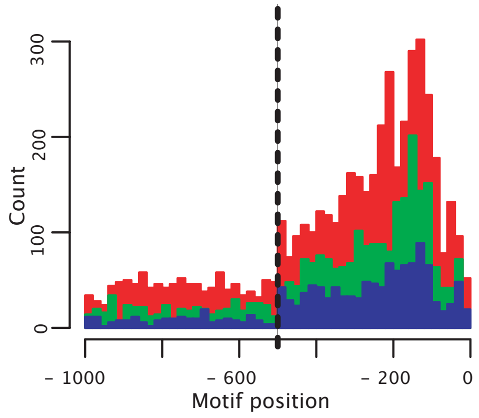

Figure 12. Histograms of the positions of motifs detected in *S. cerevisiae* biclusters, for the three motifs found in each bicluster. The distributions show a sharp peak near -150bp. The location of this peak, as well as the shape of the distribution, agrees with previous estimates of the distributions of regulatory binding sites in yeast and other organisms [82, 90, 39] and is an indication that many of the detected motifs are likely to be functional.

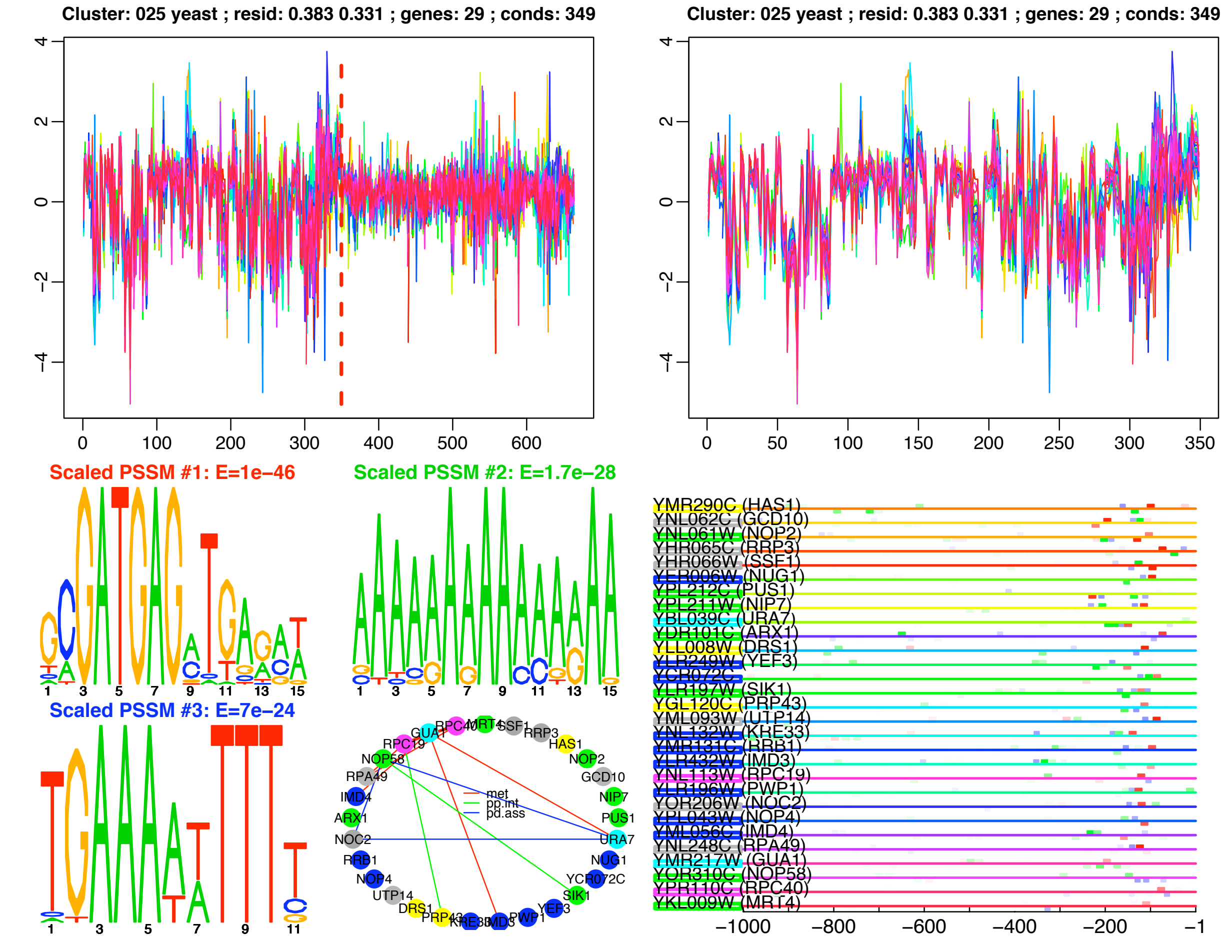

Figure 13. Ribosome bicluster from *S. cerevisiae*, containing a highly-conserved copy of the PAC motif [31] with consensus GATGAG, a known target of the Buf/Rpa regulator [59]. The motif is found at similar locations upstream of their gene coding start sites. We obtained other clusters with slight variations on the consensus of this motif (mostly in flanking residues) that could be demonstrative of the effect of small differences in binding sites on their resulting expression patterns. The PAC motif appears in close conjunction with two others: the semi-palindromic TGAAAaTTTt RPE motif and a low-complexity poly-T motif. The latter of these is often removed by low-complexity pre-filters applied by motif detection software; however it appears to be loosely-correlated in sequence position with the locations of the other two motifs. The genes in this cluster are involved in translation [19].

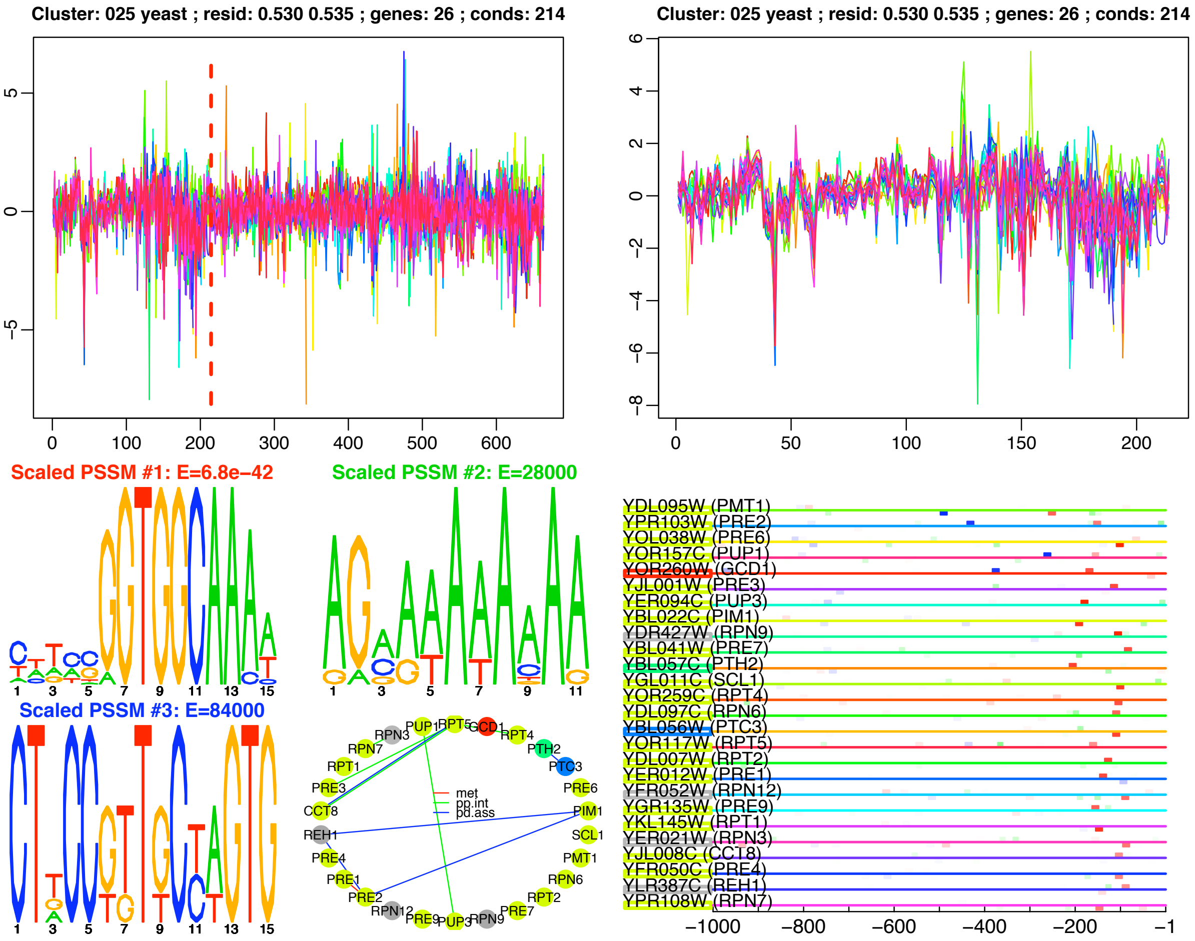

Figure 14. Proteasome bicluster from *S. cerevisiae*. The cis-regulatory motif detected for this bicluster with consensus GTGGCAAA is the well-known binding site for Rpn4 [57].

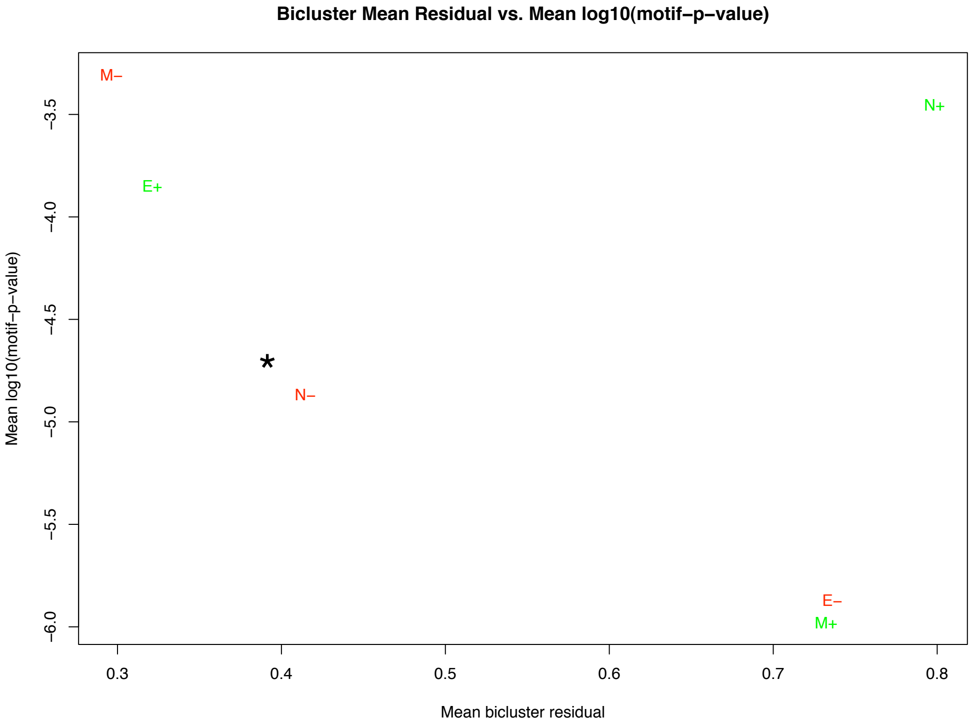

Figure 15. Mean bicluster "scores" (same as shown in Figure 6) for different runs of cMonkey with expression data (E), motifs (M) and association networks (N) individually down-weighted (-) and up-weighted (+). The canonical cMonkey run with default parameters is indicated by (*). This figure suggests that each different component of the cMonkey bicluster model is effectively regularizing the other model components, and that the default parameters result in a suitable compromise between them all.

Organism	Algorithm	k	$\langle n_{\text{gene}} \rangle$	$\langle n_{\text{experiment}} \rangle$	⟨volume⟩	⟨residual⟩	omitted[2]	f	RMSD
Halo.	cMonkey	200	18.9	150.1	2778	0.38	249	0.65	0.68
	SAMBA	196	32.4	20.2	651	0.47	540	0.12	0.93
	Cheng-Church	97	31.1	15.8	839	0.66	538	0.09	0.76
	ISA	42	86.8	10.4	880	0.58	1008	0.02	0.97
	OPSM	5	46.4	11.0	450	0.16	1860	0.03	0.98
	BIMAX[3]	134	5.8	10.2	59	1.43	1961	0.002	0.99
H. pylori	cMonkey	119	18.8	34.0	639	0.34	166	0.65	0.68
	SAMBA	32	62.8	8.3	528	0.62	174	0.21	0.86
	Cheng-Church	89	12.5	6.8	86	0.70	111	0.20	0.95
	ISA	18	16.3	4.0	65	0.54	643	0.02	0.96
	OPSM	9	52.7	7.1	374	0.25	613	0.03	0.97
	BIMAX[3]	200	18.1	4.2	76	1.95	348	0.06	0.96
E. coli	cMonkey	200	16.2	49.7	803	0.40	1037	0.37	0.91
	SAMBA	75	83.7	8.1	653	0.56	1296	0.10	0.82
	Cheng-Church	94	20.2	8.7	176	0.65	1722	0.08	0.97
	ISA	27	90.1	4.6	417	0.94	1469	0.03	0.94
	OPSM	8	47.0	10.8	505	0.27	2750	0.01	0.99
	BIMAX[3]	200	14.2	14.2	73	1.15	2061	0.02	0.96
S. cer.	cMonkey[1]	200	15.7	19.5	307	0.39	496	0.12	0.89
	SAMBA	114	61.8	16.6	1137	0.50	681	0.16	0.92
	Cheng-Church	96	34.7	16.1	559	0.79	560	0.17	0.97
	ISA	59	42.7	8.7	370	0.62	1192	0.03	0.95
	OPSM	7	47.1	12.7	599	0.11	1883	0.01	0.99
	BIMAX[3]	167	13.3	10.0	134	1.24	1757	0.01	0.98

Table 2. Bulk properties of bicluster sets from various biclustering algorithms on four different organisms. k: number of biclusters detected; $\langle n_{\text{gene}} \rangle$: mean number of genes; $\langle n_{\text{exp}} \rangle$: number of experiments; ⟨*volume*⟩: mean number of cells in the expression data matrix (genes × experiments) included in biclusters; ⟨*residual*⟩: mean bicluster mean residual [25] is a measure of bicluster coherence; *omitted*: number of genes not included in any biclusters; f: fraction of expression data matrix covered by at least one cluster; RMSD: root mean squared deviation of expression data "generated" by biclusters from the original data (see Methods for details). Bicluster sets were post-filtered as described in the Methods section. [1]The *S. cer.* cMonkey run was pre-configured to return roughly the same number of experiments per bicluster as the SAMBA run. [2]Out of a total of 2068 genes (*Halo.*), 2045 (*S. cer.*), 2936 (*E. coli*), 819 (*H. pylori*). [3]The residual measure for BIMAX is a misleading measure of cluster coherence; activation is not discriminated from repression for this algorithm.

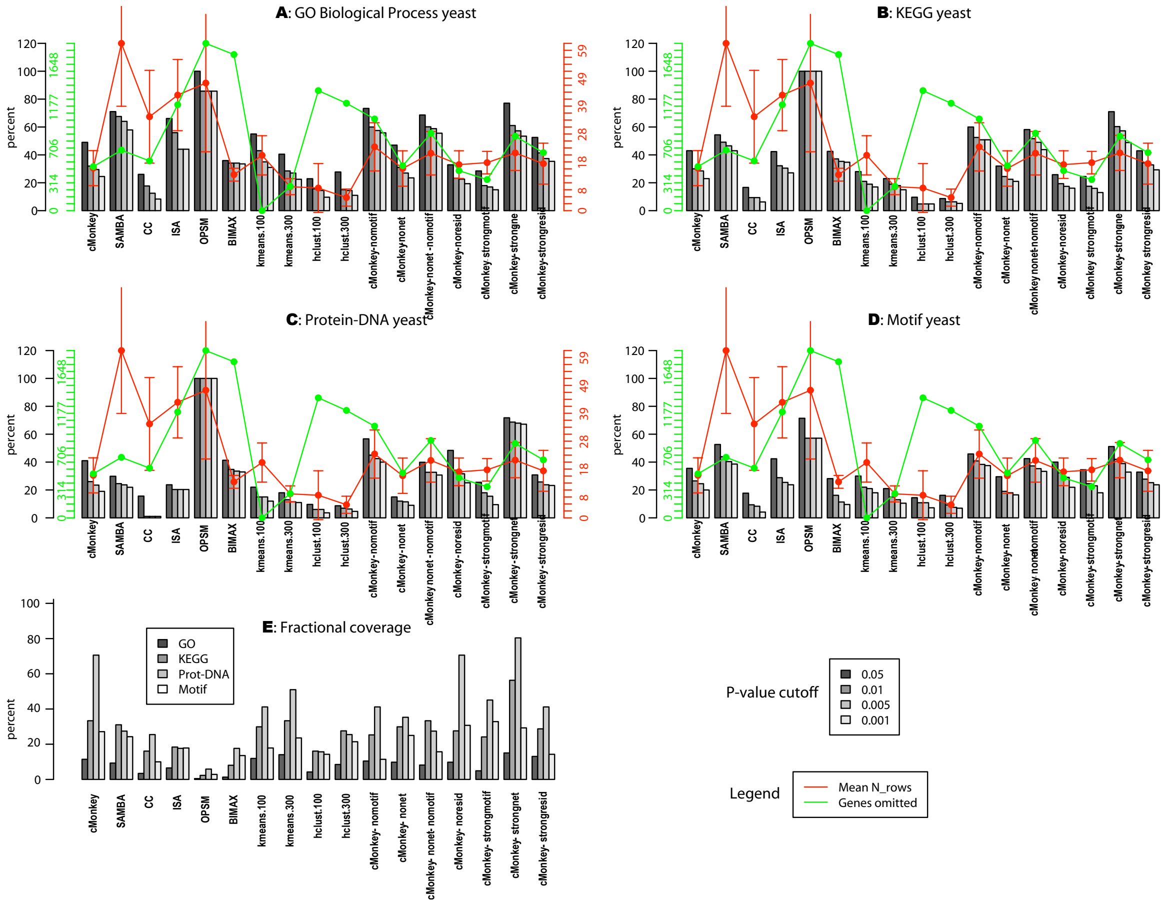

Figure 16. Sensitivity (A-D) and coverage (E) of various external measures (A: Gene Ontology biological process; B: KEGG classification; C: protein-DNA interaction groupings by transcription factor; D: matches to known transcription factor binding sites) by biclusters generated by different biclustering and clustering algorithms, for *S. cerevisiae*. The sensitivity bar plots show the fraction of bi/clusters from each set that contains a significant hit to the particular class, above the given p-value cutoff. Because there is a bias toward better sensitivity measures by larger bicluster sets with less gene coverage, the mean bicluster gene sizes (red) and number of genes omitted from any bicluster (green) have been overlaid on top of the sensitivity bar plots. The coverage plot (E) shows the fraction of all classes in each set that are significantly enriched in at least one bi/cluster at p=0.05.

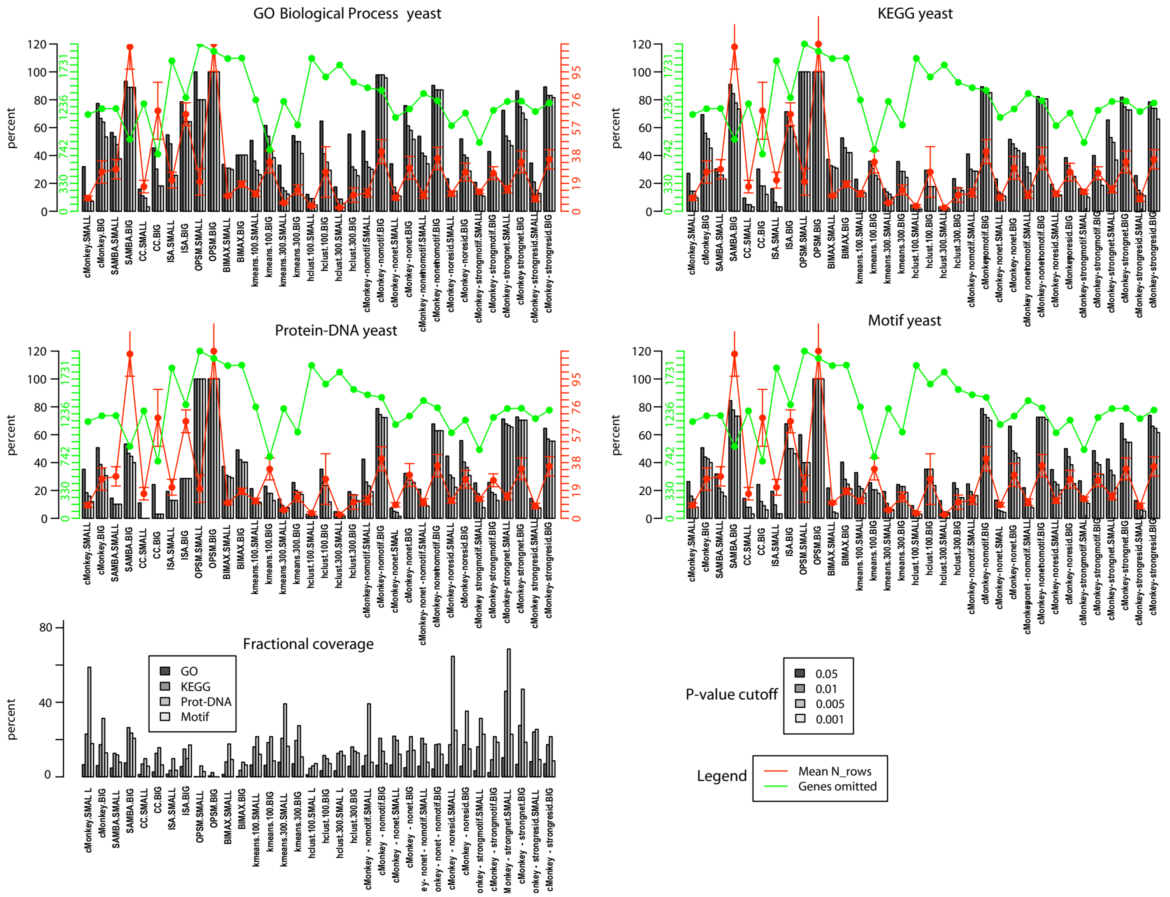

Figure 17. Same as Supplementary Figure 16, but each bi/cluster set has been split equally into two sets: the largest half ("BIG") and the smallest half ("SMALL"). This enables a more direct comparison in which the effect of bicluster size and gene coverage can be seen. For example, whereas Supplementary Figure 16 suggests that cMONKEY is not as sensitive to GO function as SAMBA and ISA biclusters, we see here that the largest cMONKEY biclusters are competitive, in terms of these measures, with the smaller SAMBA and ISA biclusters, which have roughly the same mean size. We note that OPSM appears to do better in all cases largely because it generated ~ 10 biclusters that contain $< 50\%$ of the genes in the data set. We have not been able to devise a single measure that correctly accounts for all of these discrepancies; instead we include all of this information to paint as complete a picture as possible of the different characteristics of clusters generated by various clustering and biclustering methods.

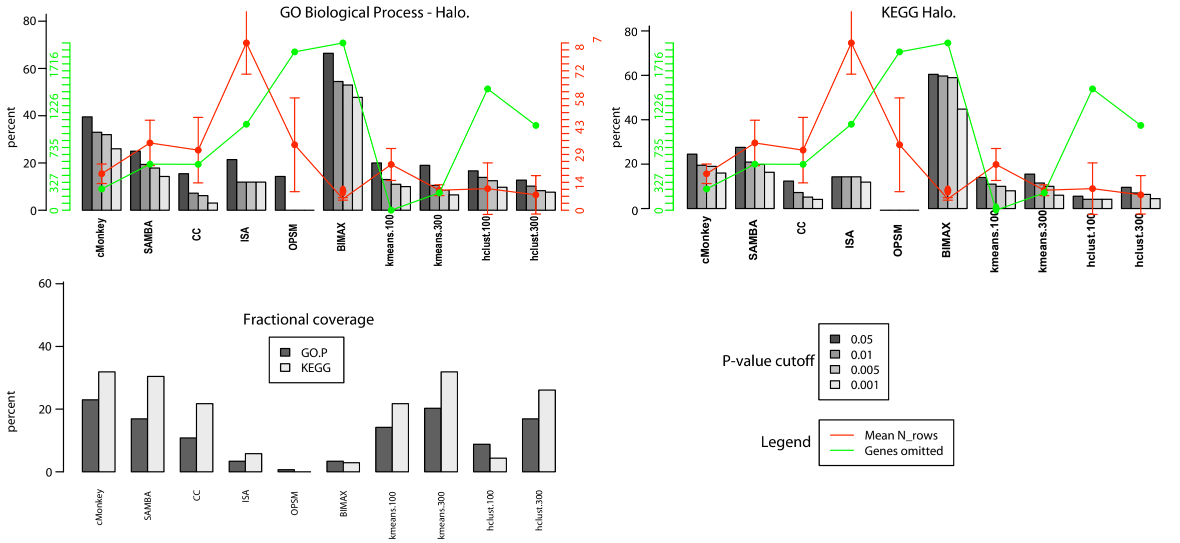

Figure 18. Same as Supplementary Figure 16, but for *Halobacterium*, rather than *S. cerevisiae*. Only cMonkey with default parameters was run here. Only GO Biological Process and KEGG comparisons are included.

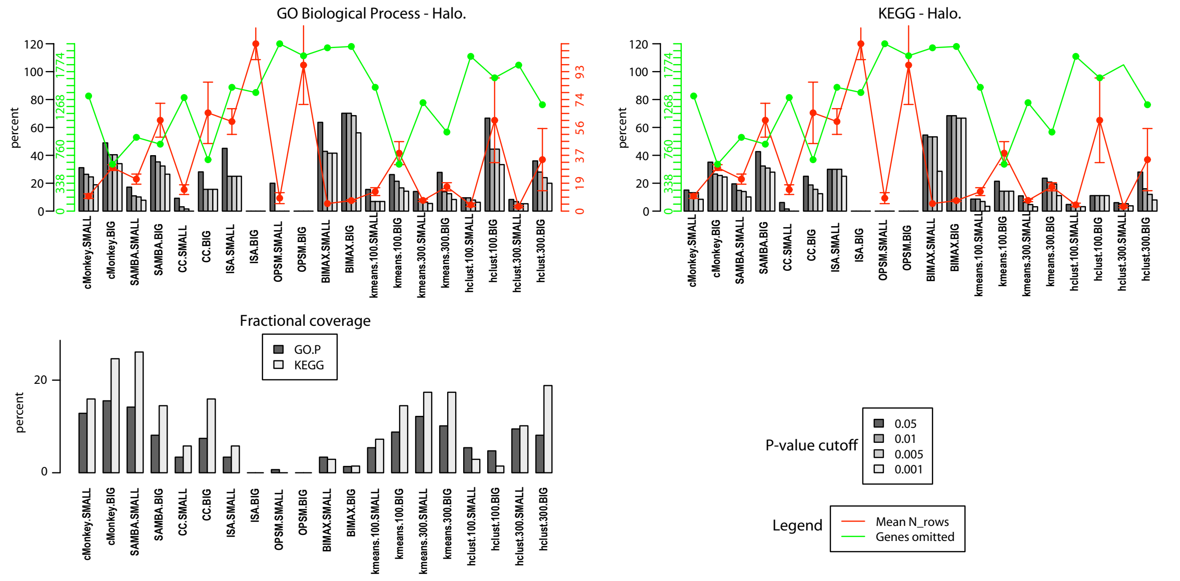

Figure 19. Same as Supplementary Figure 17, but for *Halobacterium*, rather than *S. cerevisiae*. Only cMonkey with default parameters was run here. Only GO Biological Process and KEGG comparisons are included.

Parameter	Description	Default Value
n_{iter}	Number of optimization iterations (per bicluster)	100
$k_{\max}$	Maximum number of biclusters optimized	300
r_0	Expression component weight	1.0
$s_0(\text{iter})$	Motif component weight (as a function of iteration)	0 (iter $<$ 20)<br>0 : 1.0 (iter $\geq$ 20)
q_0 (operon)	Network (operon interaction) component weight	0.3
q_0 (PP int)	Network (prot.-prot. interaction) component weight	0.3
q_0 (PD int)	Network (prot.-DNA interaction) component weight	1.0
q_0 (met.)	Network (metabolic) component weight	0.8
q_0 (PP)	Network (phylo. profile) component weight	0.5
ϵ	Parameterized systematic error in expression	0.05
v	Expected number of biclusters per gene	2
μ_k	Expected (mean) bicluster size	30
$T(\text{iter})$	Annealing temperature (as a function of iteration)	0.15 : 0.05
$n_{\text{motifs}}(\text{iter})$	Number of motifs searched (as a function of iteration)	1 (iter $<$ 45)<br>2 (iter $<$ 70)<br>3 (iter $\geq$ 70)
l_{search}	Number of upstream residues searched for motifs	250
l_{scan}	Number of upstream residues scanned for motifs	$2 \times l_{\text{search}}$
w_{motif}	Motif width (range)	6 : 22
o_{bg}	Order of background markov model used for motif search	3
Model	MEME motif model [10]	"zoops"
$E_{\max}$	Maximum motif E-value allowed	10
$P_{\max}$	Maximum motif-sequence-match P-value allowed	0.1

Parameter	*Halobacterium*	*H. pylori*	*S. cerevisiae*	*E. coli*
$k_{\max}$	300	150	600	450
n_{iter}	100	100	150	150
l_{search}	250	250	500	250
o_{bg}	3	3	5	5
μ_k	30	30	50	50

Table 3. Default cMonkey parameters (top) and species-specific values (bottom) used to obtain the results described in this paper.
